# Stereomicroscopic 3D-pattern profiling of murine and human intestinal inflammation reveals unique structural phenotypes

**DOI:** 10.1038/ncomms8577

**Published:** 2015-07-08

**Authors:** Alex Rodriguez-Palacios, Tomohiro Kodani, Lindsey Kaydo, Davide Pietropaoli, Daniele Corridoni, Scott Howell, Jeffry Katz, Wei Xin, Theresa T. Pizarro, Fabio Cominelli

**Affiliations:** 1 Division of Gastroenterology and Liver Disease, Department of Medicine, Case Western Reserve University School of Medicine, Cleveland, Ohio 44106, USA; 2 Department of Visual Sciences, Case Western Reserve University School of Medicine, Cleveland, Ohio 44106, USA; 3 Department of Digestive Health Institute, University Hospitals Case Medical Center, Cleveland, Ohio 44106, USA; 4 Department of Pathology, Case Western Reserve University School of Medicine, Cleveland, Ohio 44106, USA

## Abstract

Histology is fundamental to assess two-dimensional intestinal inflammation; however, inflammatory bowel diseases (IBDs) are often indistinguishable microscopically on the basis of mucosal biopsies. Here, we use stereomicroscopy (SM) to rapidly profile the entire intestinal topography and assess inflammation. We examine the mucosal surface of >700 mice (encompassing >16 strains and various IBD-models), create a profiling catalogue of 3D-stereomicroscopic abnormalities and demonstrate that mice with comparable histological scores display unique sub-clusters of 3D-structure-patterns of IBD pathology, which we call 3D-stereoenterotypes, and which are otherwise indiscernible histologically. We show that two ileal IBD-stereoenterotypes (‘cobblestones' versus ‘villous mini-aggregation') cluster separately within two distinct mouse lines of spontaneous ileitis, suggesting that host genetics drive unique and divergent inflammatory 3D-structural patterns in the gut. In humans, stereomicroscopy reveals ‘liquefaction' lesions and hierarchical fistulous complexes, enriched with clostridia/segmented filamentous bacteria, running under healthy mucosa in Crohn's disease. We suggest that stereomicroscopic (3D-SMAP*gut*) profiling can be easily implemented and enable the comprehensive study of inflammatory 3D structures, genetics and flora in IBD.

## Abstract (web summary)

The gut epithelium is damaged in inflammatory bowel disease, but capturing such lesions by histology can be difficult. Here, the authors use stereomicroscopy to visualize different 3D inflammatory structures and associated microbes in humans and in 16 genetic mouse models relevant to intestinal inflammation.

## Graphical abstract

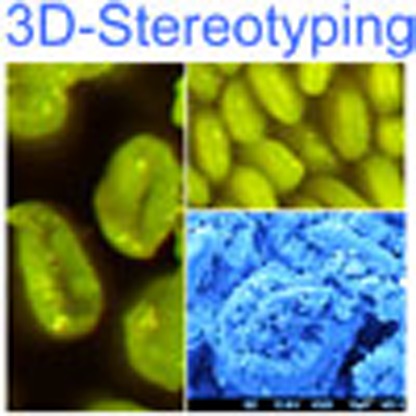

Animal models of intestinal inflammation are very popular worldwide, and are commonly used in a variety of fields. Historically, mice have been critically important in basic and translational immunology, and in infectious diseases, inflammation-associated tumorigenesis, genetics, nutrition, probiotic and autoimmunity research^1^,^2^,^3^,^4^,^5^. More recently, the role of the gut microbiome and its influence on other systems (lung, kidney, brain, behaviour) also started to be uniquely tested in these models^6^,^7^,^8^. The number of publications with ‘colitis in mice' in PubMed now surpasses the exponential growth rate of publications in ‘colitis' (∼57,000) and ‘intestine', the latter now with more than 10,000 publications per year since 2010. Despite their popularity and tremendous phenotyping potential, none of the common inducible colitis models are part of the International Mouse Phenotyping Consortium, set to characterize thousands of engineered mutations in mice^9^,^10^,^11^,^12^. The absence of chemically inducible colitis in phenotyping^9^,^10^,^11^ is perhaps because mice often have varying responses to chemicals^13^,^14^,^15^,^16^, and there are no exact methods to comprehensively quantify gastrointestinal (GI) inflammation. Herein, we report the discovery of a novel application for SM to rapidly describe the three-dimensional (3D)-structure patterns of gut mucosal health and disease phenotypes in mouse models of colitis/ileitis (∼5–60 s cm^−1^) and human inflammatory bowel disease (IBD). We report for the first time, the discovery of cobblestone lesions in the intestinal mucosa of mice, and different patterns of mucosal involvement (stereophenotypes) never reported in IBD.

IBD, including Crohn's disease (CD) and ulcerative colitis (UC), is a complex condition that affects the digestive tract in a progressive manner^17^. In humans, progressive inflammation causes thickening of the gut wall with protruding lesions resembling ‘cobblestones' over the gut mucosa. Although cobblestones are long recognized lesions of CD^18^, it remained uncertain whether cobblestone formation occurs in any animal model of IBD. With the emerging need to discover novel therapies to downregulate intestinal inflammation and promote mucosal healing, it is critical to highlight there are no methods to comprehensively evaluate the entire gut mucosal surface and quantify its response to therapies. Historically, most of our knowledge regarding intestinal pathology has been derived from thin-tissue sections reflecting changes transmurally (across the gut wall). With exceptions^18^, it is assumed that gut inflammation occurs topographically at random, and that an average histological score represents the overall GI condition. Histological scores of inflammation, based on two-dimensional (2D) representations and integer numbers, describe, however, mostly cellular abnormalities. Complementary, endoscopy *in vivo* has facilitated the assessment of intestinal health, but its routine experimental application remains limited to the distal gut.

As a complementary tool to histology and endoscopy, herein we propose the routine use of SM to reproducibly assess the spatial appearance of the gut mucosa *ex vivo*. Invented in the 1800s, SM is widely used during *in vitro* fertilization, microsurgery and microcircuitry^19^, but remains scarcely used in GI research (Supplementary Fig. 1). In SM, light reflected from the surface of a thick specimen, via two independent nonparaxial optical paths, produces in the observer's brain a magnified live 3D-stereoscopic representation of the specimen's surface. Resembling the low-power magnification images of scanning electron microscopy (SEM), we discovered that stereomicroscopy (SM) has tremendous potential as a routine diagnostic tool for real-time topographical analysis of the GI tract.

Herein, we: (i) compare the performance of endoscopy, histology and SM, (ii) describe the development, SEM-validation and implementation of a 3D-SM Assessment and Pattern Profiling (3D-SMAP*gut*) protocol to map/quantify the intestinal surface architecture and functional relevance in mice and (iii) illustrate the value of SM for the assessment of intestinal specimens from humans with CD.

## Results

### Distinctive genetics of mice with CD-like ileitis

Because SM provides information in real time and correlates well locally with histology in farm animal^20^ and human gut biopsies^21^, we developed a comprehensive protocol to profile the 3D architecture of the entire mucosal surface using SM and mice as a model system to improve our understanding of gut health variability across genetic backgrounds^22^,^23^. Since ileitis (inflammation of ileum) and cobblestones are common in CD, we started examining mouse strains known for developing spontaneous ileitis, SAMP1/YitFc (SAMP) and C57BL/6J(B6)^TNFdeltaARE/+^ (TNF^**ARE**^) (MP:0009482), and ileitis-free controls AKR/J (AKR) and B6 mice. We then analysed other strains and models of inducible and spontaneous colitis in mice, as well as surgical specimens from humans with CD. As CD models, SAMP and TNF^ARE^ mice have divergent genetic backgrounds, but develop progressive ileitis with 100% penetrance by 30 weeks of age^24^. SAMP originated from inbreeding of AKR with presumptive contamination by B6 and other strains^24^. TNF^ARE^ carry a deletion in the AU-rich elements (ARE) of the tumour necrosis factor (TNF) gene causing elevated concentrations of proinflammatory TNF, which leads to progressive arthritis and ileitis^25^.

### Unique 3D patterns of IBD structure in mice

**Figure 1:**
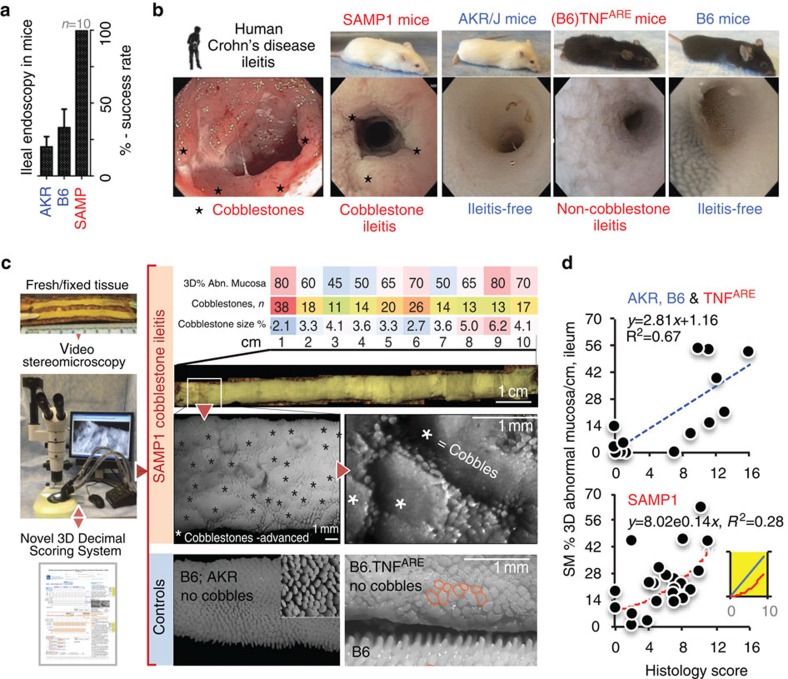
Stereomicroscopy allows the rapid topographical analysis of the entire intestinal mucosa in mice. (**a**) Although small intestinal endoscopy is possible in mice, complications in healthy control mice due to tissue friability make endoscopy suboptimal/biased for routine murine GI examination. (**b**) Endoscopic images of terminal ileum from humans, ileitis-prone SAMP1 and TNF^ARE^ mice and ileitis-free genetic controls AKR/J and B6. Notice the similarity of obstructive cobblestone ileitis for humans and SAMP mice, and distinct endoscopic pattern in TNF^ARE^. (**c**) To increase our understanding of intestinal diseases, we developed a 3D-SM Assessment and Pattern Profiling protocol (3D-SMAP*gut*) for fresh and fixed postmortem specimens to evaluate the mucosal architecture of both small and large intestine. The system characterizes abnormal/normal mucosa, cm by cm, using a reference catalogue of SM lesions, validated parameters (see Methods and Supplementary Figs 8–10). SM allowed the discovery of previously unknown ‘cobblestone' lesions in animals/mice. (**d**) Correlation between ileal SM (3D% of abnormal mucosa, 3D%-AbMuc) and traditional histological inflammation scores in mice. Notice histological scores plateau at 12 in SAMP (curved exponential fitting) compared with AKR and B6 (linear fit; higher R^2^). Inset line plot, simulation of SM 3D%AbMuc represented mathematically as circles whose cobblestone areas (area=*πr*^2^, red curved line) grow exponentially as their diameter increases. Radius, surrogate for histological score on intestine's longitudinal axis, increases as cobblestones grow (straight line, *y*=*r*). Lower *R*^2^ indicates larger variability between SM and histology in SAMP. SM-guided dissection of lesions decreases variability and increases biological/statistical study power.

We realized the diagnostic potential of SM during studies with endoscopy^26^, when we reported a scoring protocol based on a novel decimal-identifier system to integrate colitis and colonic cancer in mice, and an endoscopic technique under general anaesthesia to examine the ileum (ileoscopy) *in vivo*^26^. Although ileoscopy was possible as a terminal procedure in ileitis-free mice, there were complications due to intestinal tears and friability. Herein, we examined ileitis-prone SAMP and TNF^ARE^ mice compared with controls using the same terminal postmortem procedure. In regard to frequency of complications, B6, AKR and TNF^ARE^ mice had more complications than SAMP mice (Fig. 1a), indicating that the inflamed/thickened wall of the SAMP ileum prevents endoscopy-associated tears, and that ileoscopy would therefore be a biased technique to examine the small intestine. However, despite this bias, the images obtained indicated that SAMP and TNF^ARE^ mice would have distinct 3D patterns of IBD architecture, suggesting for the first time that host genetics could drive unique spatial inflammatory patterns in the gut. Because the assessment of entire mucosal surfaces would not be routinely possible in live mice with currently available endoscopic technologies (with the exception of the distal 2–3 cm of colon), we used SM as a bias-free technique to test such hypothesis postmortem. Remarkably, all SAMP mice (with/without commensal gut flora) examined at 30 weeks of age (equivalent to ∼20 human years^22^,^27^,^28^) had unique endoscopic lesions resembling CD's typical human ‘cobblestone' lesions (Fig. 1b,c). In contrast, TNF^ARE^ had enlarged and distorted villi but no cobblestone formation; further supporting that SAMP and TNF^ARE^ have previously unknown divergent IBD 3D-structural phenotypes (stereophenotypes) and spatial pathophysiology (Fig. 1c and Supplementary Fig. 2).

### SM reveals mucosal 3D complexity

To evaluate the role of SM for global assessment, we then examined the entire digestive mucosal surface in ileitis-prone and control mice. In addition to cobblestones, SM allowed the recognition of microscopic features of IBD-associated periodontal disease^29^, retropharyngeal abscesses and gastric/intestinal mucosal haemorrhages. Remarkably, novel patterns of focal lesions, including linear ulcers and villous tip erosions were also identified, and confirmed not to be artificially magnified due to tissue degradation during ethanol post-fixation storage, in the small intestine of control mice. The clinical relevance of focal lesions in control mouse strains regarded as IBD-free is unknown, as it is the relevance of sporadic lesions in asymptomatic humans. A mucosal immunological role of several lesions is however expected because SEM revealed drastic epithelial barrier disruption in SM-targeted/dissected lesions. Of practical relevance, animals euthanized and left at room temperature had major gut mucosal SM changes due to intestinal autolysis within 2–3 h of euthanasia. Of mechanistic relevance, most IBD-prone mice having one lesion had concurrently at least another type of 3D anomaly, highlighting the need to (i) assess the entire mucosal surface during experimentation and (ii) consider evaluating specific (target) 3D-pattern structures in IBD research. Of diagnostic relevance, experts regarded some focal abnormalities as histologically sound or as artifacts until further SM and SEM images were examined. Underappreciating the histological relevance of lesions based on the diameter of focal lesions (<0.5 mm, few villi) compared with the specimen's length (∼10 cm) was common; however, SM revealed that certain lesion types are oriented perpendicularly to the longitudinal gut axis, indicating that such lesions often may go unnoticed in pathology (Supplementary Figs 3–7). Traditional IBD experimentation in mice could therefore be partly confounded by the unrecognized presence of concurrent 3D-divergent co-pathologies. Understanding their possible 3D and mechanistic interdependence will be critical in addressing IBD.

### Structure-pattern profiling with 3D-SMAP*gut*

To reproducibly profile the detailed appearance of the GI tract, additional mice were examined to create a comprehensive SM catalogue of histologically and SEM-validated 3D-intestinal abnormalities. The catalogue now represents >700 mice, >16 inbred strains, and various murine models of acute/chronic intestinal inflammation and infection (Table 1). Ranked by morphology and clinical relevance, the abnormalities were grouped into 10 ordinal lesion categories (0–9) for use as decimal identifiers, following our multidimensional scoring rationale^26^. To determine lesion co-occurrence and spatial distribution patterns, we designed a system (3D-SMAP*gut*) and a register form to capture qualitative and quantitative data, cm by cm (Fig. 1c and Supplementary Fig. 8).

A 3D-SMAP*gut* score contains information regarding the extent of abnormal mucosa and the lesions that account for it. The scale developed for the small intestine ranges from 0.000 to 1,000.987. A score of 0 means healthy mucosa, while 456.789 indicates that the segment examined has an average of 45.6% of abnormal mucosa per linear (tubular) cm of gut examined, while decimals 789 indicate that the abnormality is due to lesion categories 7, 8 and 9 in order of prevalence. For the colon, which lack villi, we developed a slightly different system (SM-colon score) accounting for colon shortening during colitis, and anatomical differences between the distal and proximal colon (see Methods and Supplementary Figs 9–11). Of relevance, distinct 3D-SM appearances were noticed for DSS-colitis, AOM/DSS tumour formation and for a variety of focal lesion not previously recognized in the colon of adult mice, including lymphoid aphthous ulcers, ‘miliary' lesions, marked distal colonic ‘corrugation' and mucosal nodules in proximal colon folds (Supplementary Fig. 7).

### Divergent stereophenotypes in experimental CD

Taking the percentage of 3D abnormal ileal mucosa from AKR, B6, TNF^ARE^ and SAMP mice (*n*=48), we quantified the discriminatory potential of 3D-SMAP by determining that the correlation between SM and histology scores improved by data stratification based on presence/absence of cobblestones. Combined, the correlation coefficient of determination *R*^2^ for all mice/strains was 0.52, which improved its linearity (*R*^2^=0.67) after removing the SAMP data. In contrast, SAMP data was not linear (*R*^2^=0.28), but exponential with a plateau of histological scores as the SM scores increase (see distribution/equations in Fig. 1d).

Conceptually, SM assesses lesions as circumferential areas that grow on the gut surface, while histology assesses lesions as lines that grow along the specimen's longitudinal axis (Fig. 1d insert). Analysis of five indexes composing our histological scores indicated TNF^ARE^ had more severe intestinal inflammation (i.e., higher scores) than SAMP, suggesting TNF^ARE^ could be mistaken as a better IBD model without the aid of SM (Supplementary Fig. 12). However, 3D-SM pattern profiling of gut inflammation revealed that SAMP and TNF^ARE^ models have divergent 3D-structural inflammatory phenotypes (SAMP having CD-like ‘cobblestone' lesions present throughout the small intestine with increased density in the ileum, and TNF^ARE^ having ‘villous mini-aggregates' with apparent mucosal corrugation restricted to the terminal ileum) making both models equally valuable to investigate why inflammation in IBD is chronic and self-perpetuating in nature, and regionally variable in humans (see 3D-stereoenterotypes, Supplementary Movie 1).

### Disease-severity bimodality and SM-functional target analysis

**Figure 2:**
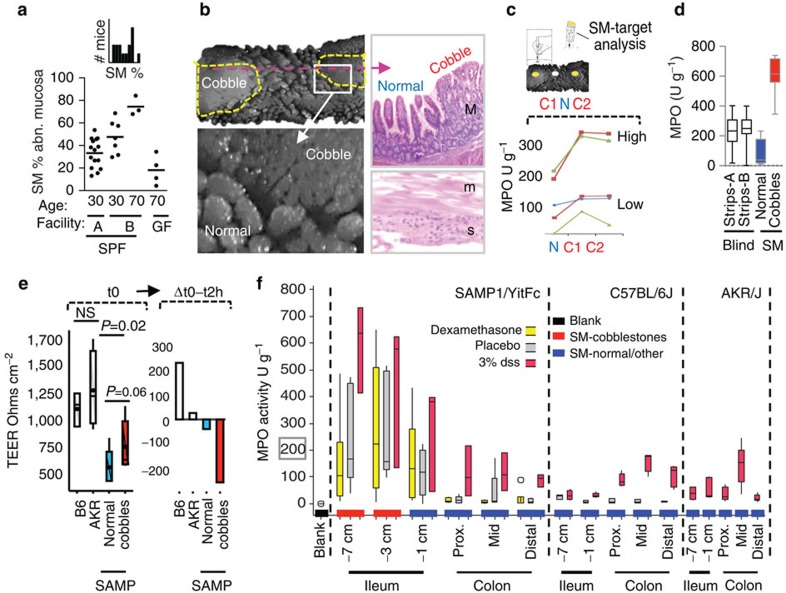
SM-target normal–abnormal paired sampling for functional analysis of multifocal ileitis in mice. (**a**) SM differentiated cobblestone-ileitis disease severity in SAMP mice as a function of age, housing facility and presence of commensal gut flora (GF, germ-free; SPF, specific pathogen free). SM also revealed disease-severity bimodal distribution (inset histogram—facility A, subpopulations of mice ∼50% each with significant severity differences). Facility A and B mice are littermates born in facility A but split as cohort and transferred to facility B at weaning, 4-week-old. (**b**) Histology showed SM cobblestones have discrete (yes/no) morphological features at villous level that allows 3D-SMAP*gut* to precisely quantify the extent of abnormal mucosa. Notice transmural histological inflammation and cellularity in serosa. M, mucosa; m, muscular layers; s, serosa. (**c**) MPO activity in SM paired cobblestones-normal mucosa of co-housed littermates showed increased MPO in cobblestones, and significant overall reactivity in 2/5 of mice. (**d**) SM-target analysis provides more accurate information compared with blindly collected strip-longitudinal intestinal samples. SM-‘within-mouse case-control' sampling will allow the identification of focal modulating factors for cobblestone formation and other SM-lesion types in IBD. Strips-A and -B represent two groups of six to eight mice; normal-cobbles, represent a third group sampled using SM. (**e**) TEER *ex vivo* analysis on SM-dissected cobblestones and SM-normal tissues (Mann–Whitney, *n*=4–5 per group). (**f**) Intervention experiments with dexamethasone and DSS showed that SM-target MPO analysis predictably enabled the ability to quantify and differentiate treatment response to therapies across mouse strains and IBD murine models (GLM regression; *n*=4–8 per group; reproducible in five other comparable experiments). Boxplots in figure display interquartile range boxes, median, min and max values; symbol inside box indicates mean.

Studying SAMP cobblestone ileitis (%), SM also detected in cross-sectional studies, disease differences as mice aged, differences between animal facilities, disease amelioration in germ-free conditions as expected^24^; and of novelty, a bimodal population distribution of disease severity (that is, not Gaussian/single bell-shaped; but with two side-by-side-bell-shaped peaks with ∼50% of mice suffering advanced disease), suggesting the presence of underlying epigenetic factors modulating disease severity within the same genetic line (Fig. 2a), littermates and co-housed mice.

SM microdissection of within-mouse paired intestinal samples was then used in functional assays of SM-target-dissected fresh tissues following an in-house validated protocol^30^. Myeloperoxidase (MPO) is a potent antibacterial oxygen-free-radical-producing enzyme present in cytoplasmic vesicles from neutrophils and leukocytes^31^. By using MPO as a sensitive biochemical marker for inflammation, SM paired samples confirmed that cobblestones had significantly more MPO compared with normal areas, and that the disease-severity bimodality concept persisted biochemically (*t*-test *P*<0.001; Fig. 2b,c). To determine whether SM could detect differences in another mouse model that differed genetically in only one theoretically relevant IBD gene (that is, non-TNF^ARE^), we developed and tested SAMP mice with a single homozygous NOD2-knockout mutation^32^. Of statistical value, such experiment showed that SM and MPO could differentiate wild types from knockouts only with SM-target sampling (cobblestones versus normal), and not with single-longitudinal-strip blinded sampling (Fig. 2d). Differences between wild-type and NOD2 knockouts have also been SM-resolved for other markers^32^, indicating that 3D-SMAP profiling facilitates the phenotyping of genetically engineered mice.

SM target sampling was also used for transmembrane epithelial electric resistance (TEER) assays *ex vivo*. CD has been associated with the hypothesis of a membrane permeability defect in the intestinal epithelium, and SAMP mice have a similar defect^33^,^34^. Using normal–abnormal SM sampling, we discovered for the first time that the TEER defect known in SAMP is profoundly pronounced in cobblestones areas (Fig. 2e). SM profiling and paired sampling facilitates elucidating focal mechanisms of IBD severity.

### Faecal flora homogenization before SM studies

Because there is evidence of cage-to-cage microbiome variability, intervention experiments with 30-week-old SAMP mice were also conducted in a well-controlled fashion^30^ to determine whether 3D-SMAP could identify treatment effects in cobblestone ileitis and DSS-colitis. Following a period of co-habitation and faecal transplant manipulations to promote inter-cage-strain gut flora homogenization before experimentation^30^ to minimize confounding effects due to flora functional divergence (drift or shift in mice raised separately for 30 weeks; or for generations in knockout versus wild-type colonies), animals were treated with either (i) anti-inflammatory dexamethasone (5 mg kg^−1^, every 24 hours, intraperitoneal, 7 days), (ii) pro-inflammatory 3% DSS in drinking water to induce chemical colitis (5 days, with a phosphate-buffered saline (PBS) intraperitoneal injection) or (iii) placebo (PBS daily injections). After 7 days, ilea and colons were harvested for SM, histology and functional assays. Treatment interventions exerted their effects as expected (DSS increases, while dexamethasone decreases, histological inflammation versus placebo; adjusted *P*<0.05, *n*=25). The correlation between SM and histology depends on the effect induced by the treatment. For ileitis, all data best fitted an exponential model (*R*^2^=0.89, *y*=6.72 × e0.19*x*; versus linear model *R*^2^=0.54, *y*=2.83x+4.44) as shown earlier. For colons, the correlation was stronger and linear as changes in SM scores were high in DSS-colitis, and intermediate or low for dexamethasone and placebo treatments (*R*^2^=0.91, *y*=8.03*x*+13.37). SM-colon scores not only revealed treatment differences, but also patterns of lesion distribution regionally (mid-colon more affected with DSS), indicating that study errors are expected when different intestinal segments are subjectively recommended^35^,^36^ for different diagnostic assays.

### MPO and biochemically weighted 3D-SMAP*gut*

Although MPO activity is widely used as a marker of inflammation^35^,^36^, the test is known for ample variability. On the basis of SM and an improved MPO estimation methodology for SM-microdissected tissues using beat-beating homogenization and micro-well formats^30^, we hypothesized that such variability and its clinical value would be improved if tissues were sampled according to 3D-morphology. To evaluate the correlation between SM, MPO and gene expression, six fresh intestinal samples were collected per mouse (ileum/colon) to conduct between and within-mouse paired analysis. As expected, the 3D appearance of the mucosa corresponded remarkably well to the amount of MPO when samples as little as 5 mg per test were SM-dissected for testing. Cobblestone-affected ilea had more MPO than adjacent normal mucosa, which was higher than in the affected mucosa of B6 and AKR mice during DSS treatment, contextualizing the 3D-SMAP robustness and the severe inflammation that occurs chronically in SAMP ilea (Fig. 2f). The collection of smaller samples for miniaturized assays is possible (as in fertilization medicine^19^), but depends on purpose and sampling strategy.

**Figure 3:**
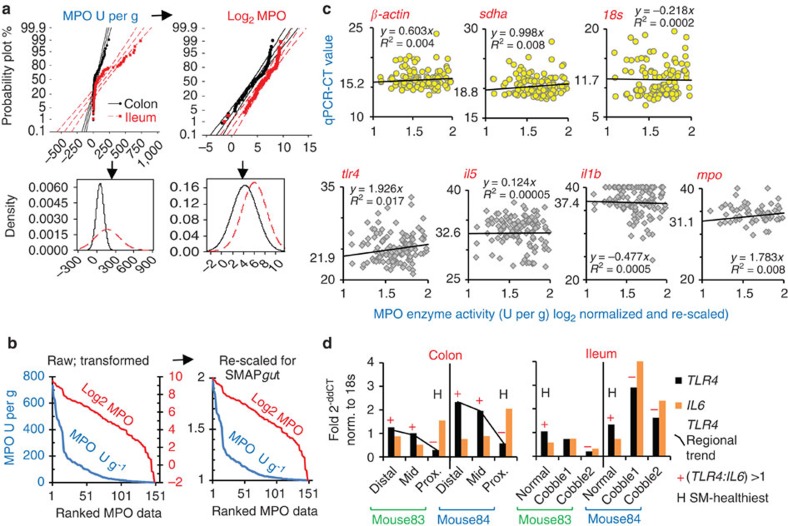
Re-scaling of MPO data for weighted-3D-SAMP*gut* and gene expression analyses in mice. (**a**) MPO data from Fig. 2f is not normally distributed (*P*>0.1) and therefore not comparable for weighted analysis across SM-dissected tissues. Log-transformation allowed comparable normalization for ileum and colon (*n*=150 samples; B6, AKR, SAMP). (**b**) Re-scaling of MPO activity for adjustment of SM scores (percentage of abnormal mucosa). Log-transformation favours differentiation of samples with low MPO (steepest, below curve shoulder ∼200 U g^−1^). (**c**) Raw qPCR-CT gene expression values correlation plots versus re-scaled MPO activity. In β-actin panel (narrowest variability), *y*=0.603*x* represents linear correlation slope (for each unit of increase in re-scaled-MPO, β-actin increased by 0.603 units); value 15.2 indicates intercept. Low *R*^2^ indicates variability departing from fitted line cannot be explained by equation or MPO activity alone. (**d**) Example of SM-targeted 2^−ddCT^-fold analysis of *il6* and *tlr4* genes (six samples per mouse) for two randomly selected dexamethasone-treated mice. Note reproducible regional expression profile variability within colon (*tlr4:il6* ratio is highest distally, but lowest proximally), suggesting SM-guided studies are best by normal–abnormal paired sampling within the same region. Paired SM sampling of ileum shows also a pattern: high *tlr4:il6* ratio in normal areas, but low in cobblestones. Paired sampling may reveal biologically relevant spatial/structural differences within the same mouse strain: for example, *il6* is overexpressed in proximal colon, which is less inflamed in DSS-colitis, but also in small intestinal cobblestones which are markedly inflamed in SAMP ileitis. IL6 varies (anti/proinflammatory) in the same mouse strain depending on location and SM-lesion structure.

Because the percentage of 3D abnormal mucosa may not change (heal) with some treatments, but inflammation could decrease transmurally, we established that, if desired, the percentage of abnormal mucosa could be mathematically adjusted with its MPO value to create a biochemistry-weighted 3D-SMAP system to differentiate intestinal responses using a biochemical marker. This approach is useful if two mice had the same percentage of abnormal mucosa. We discovered that differentiation is possible by multiplying the percentage of abnormal (and normal) mucosa by their respective MPO values after re-scaling the Log_2_-normalized MPO data (Fig. 3a,b). Re-scaled MPO (ranging between 1 and 2, minimum and maximum for given laboratory/experiment, see Methods) doubles the SM percentage value when MPOs are highest (value 2), or has no impact when MPOs are lowest (value 1). Although the addition of the MPO-weighted SM percentage areas is simple and provides a comprehensive linearized measurement of disease extent and inflammation severity (in arbitrary scale, 0–200), analysis of data is also possible separately using multivariate statistics. The MPO-weighted SM concept may apply to other biochemical markers, and it is a simplified integrated multivariable attempt to aid researchers with intestinal phenotyping.

### Reference genes for SM-gene expression analysis

To decrease variability and enhance study power for differential gene expression (DGE) analyses in GI research, we determined how mucosal immunity-related gene expression varied across distinct SM mucosal areas (toll-like receptor 4 (TLR4), receptor for bacterial sensing; interleukin-1β, proinflammatory; IL-5, eosinophil chemoattractant; IL-6, pro- and anti-inflammatory; MPO^37^), and examined three candidate reference (housekeeping) genes selected from humans^38^. Under highly reproducible and paralleled experimentation, real-time quantitative (q)PCR-CT values from 252 SM-dissected intestinal paired samples from mice in Fig. 2f were used to quantify gene mRNA variability (Supplementary Fig. 11). Our goal was to identify the most stable (less variable) reference gene across a range of inflammatory scenarios using qPCR-CT data on SM-dissected tissues. Re-scaled MPO was used to rank the inflammation severity in ileal/colonic tissues across three mouse strains after pro- or anti-inflammatory treatments (that is, DSS or dexamethasone). From three housekeeping genes selected from human GI cancer and inflammation studies^38^, the β-actin (cytoskeleton) and *sdha* (mitochondrial) genes were very stable across assays and samples, compared with highly variable *18S* (ribosomal; Fig. 3c and Table 2).

With respect to treatment, all housekeeping genes showed higher expression (lower CTs) during DSS-colitis (Table 2). As expected^39^,^40^, expression differences also existed between mouse strains, suggesting that SM-‘within-mouse case–control' sampling would be ideal to identify focal epigenetic factors that modulate intestinal inflammation; especially, when genetic mouse lines respond differently to treatments^13^,^14^,^15^,^16^ (Table 2). Of relevance, the ability for *tlr4* and *il5* to differentiate treatment responses was significantly enhanced when analysis was stratified within mouse according to SM-lesion category. The relevance of DGE SM-target ‘within-mouse' fold-change analysis is illustrated with *18S* reference gene for *tlr4* and *il*6 (see within-segment variability in Fig. 3d); however, we suggest that β-actin and the separate use of *sdha* should be used to reproducibly test DGE network findings. Recently, we identified SM-target DGE benefits assessing the mutational effect in SAMP-NOD2^−/−^ knockout mice^32^. SM-targeted analysis of paired tissues within mouse is ideal to control for regional variability to study focal 3D inflammatory IBD structures.

### SM images for visualization and spatial analysis

**Figure 4:**
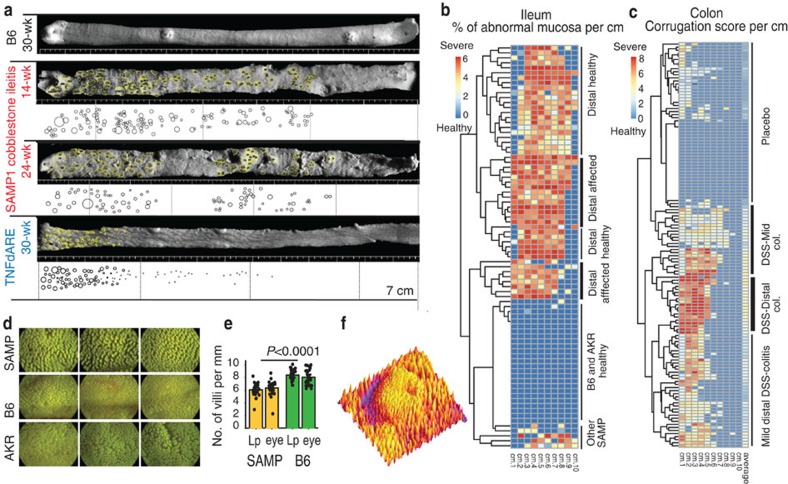
Data derived from 3D-SAMP*gut* is suitable for spatial visualization and ImageJ software analysis. Ileo-cecal junction to the left, light from the right. (**a**) Bubble plot analysis of ‘cobblestone areas' in SAMP mice contrast the marked proximad gradient of ‘villous-fusion' pattern observed in TNF^ARE^ ileitis (Supplementary Fig. 13). Note the skip/healthy area in 24-week (wk) SAMP mouse. (**b**) Hierarchical cluster (unsupervised, Euclidean) analysis of SM % abnormal areas, cm by cm, in the last 10 cm of small intestine (ileum of SAMP and control mice, *n*=135), or (**c**) colon (various strains with and without DSS treatment, *n*=214). Log_2_ transformed data. Note regional patterns of intestinal inflammation, and bimodal distribution of lesions in the last cm of small intestine (cm1) at the population level, and the presence of mouse subpopulations that are more affected in the mid-colon, while others are affected distally. With the exception of mdr1a^−/−^ adult mice (Table 1), which had pan-colitis, proximal colitis was infrequent. Heat maps, built using public-domain scripts in R (www.r-project.org/), are amenable to larger data sets (**d**) In addition to the analysis of abnormal areas, SM images also allowed the rapid analysis of intestinal villi in remaining seemingly ‘normal/unaffected' areas. (**e**) For villous count analysis, ImageJ line plots (Lp) facilitated the enumeration of villi, which was similar to counting villi by eye (*n*=30 per strain, *t*-test *P*>0.2). (**f**) 3D visualization of the mucosal surface of an ileal cobblestone structure interpreted with ImageJ.

**Figure 5:**
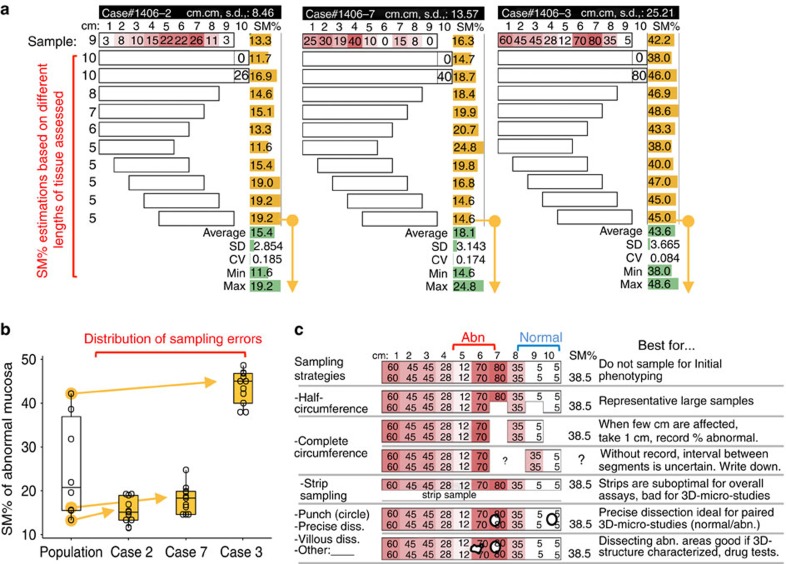
Effect of intestinal segment length on scoring variability and sampling strategies for functional assays. (**a**) Effect of length and location of the examined segment on the SM percentage of abnormal mucosa in the small intestine tested from three consultation mouse cases. Length for group was 7–9 cm; ideal is 10 cm. Zero and max values were used in calculation for cm10. Note standard deviation (s.d.) and coefficient of variations (CV=s.d./mean). (**b**) Contextualization of distribution of the estimated sampling errors for mice in **a** with respect to their actual experimental (population) group from which animals were randomly chosen. Notice that distribution variability (sampling errors) may prevent the experimenter to statistically detect small treatment effects if sampling is not systematic and if animals had mild/moderate disease (case 2 versus case 7). Boxplots display interquartile ranges, median, min and max values. (**c**) Graphical illustration of various stereomicroscopic sampling strategy types and suggested best-observed uses. The size of microdissected samples and their functional reproducibility using mRNA qPCR with reverse transcription (qRT–PCR) gene expression data are presented in Supplementary Fig. 11.

To analyse the variable patterns of SM abnormality distribution, we analysed SM photographs using ImageJ public-domain software (imagej.nih.gov/ij/). We measured single cobblestone lesions and plotted the areas for visualization and circular dimension analysis. Figure 4a shows the spatial distribution of lesions in the last 7 cm of small intestine of four mice to highlight the unique clustered regional pattern of cobblestone lesion distribution in SAMP, and its contrast to TNF^ARE^ mice. Heat-map cluster analysis of the percentage of abnormal mucosa in ileum, and the ‘SM-colon score' visually shows the presence of subpopulations of mice grouped according to patterns of spatial inflammation distribution. Intriguingly, half of SAMP mice have the last centimetre of ileum free of cobblestone formation, and there was limited evidence of proximal colitis (Fig. 4b,c). In addition to studying abnormal areas, SM imaging allowed the study of villi in seemingly normal mucosa. Line plot analysis of intestinal villous density shows SAMP had lower villous density compared with control mice, suggesting SAMP mice have increased lamina propria cellularity. This highlights the unprecedented need to study 3D-inflammation structures at the villous level; and ImageJ is suitable for 3D-SMAP (Fig. 4d–f). To further standardize SM-derived data, Fig. 5 illustrates the effect of length of the intestinal segment harvested for 3D-SMAP on scoring variability, and SM-target sampling strategies for functional assays. The preparation of printed paired SM images for stereoscopic vision is also possible as it is required in structural biology^30^.

### Minimal training for implementation

To quantify the 3D-SMAP scoring reproducibility and estimate its implementation feasibility, we first created a 10-min training session on how to estimate the percentage of abnormal mucosa, and a set of 14 SM images, which were presented to an audience of 10 untrained technicians/students for immediate testing. During training, observers were requested to engage in a mental process to add areas affected by cobblestones while subtracting areas affected by other SM lesions/artifacts, using a 1 mm bar as reference. Independent estimations demonstrated that the scoring methodology was intuitive as it was highly reproducible among novice-trained observers. Compared with instructor and ‘gold-standard' ImageJ, 2/10 of observers made highly precise estimations suggesting the process is highly intuitive and easy to be implemented. Despite inter-observer variability (<18% due to overestimation, correctable with training, Mann-Whitney *P*=0.0006), all participants agreed on their impression of disease severity in all cases (interclass *R*^2^>0.90, Supplementary Fig. 14). We then quantified the observers' ability to differentiate among SM-lesion categories. Presented with a collection of 13 questions representing large and small intestinal images and SM lesions, eight observers classified the lesions correctly in 81±11% of cases after 40-min lecture image-based training (Supplementary Fig. 15). To enhance inter-laboratory comparability, we determined that SM videos are an effective form of data archival, rapid reproducible assessment (1–15 min), and data sharing over the Internet. If scoring was implemented and offered through centralized laboratories, SM videos and reproducibility would facilitate large-scale phenotyping projects. To facilitate training implementation, the teaching and learning modules designed in this study were prepared using the figures and Supplementary Materials provided in the present manuscript, with images from mouse clinical cases for testing.

### SM in human IBDs reveals intramural pathogenic gut flora

**Figure 6:**
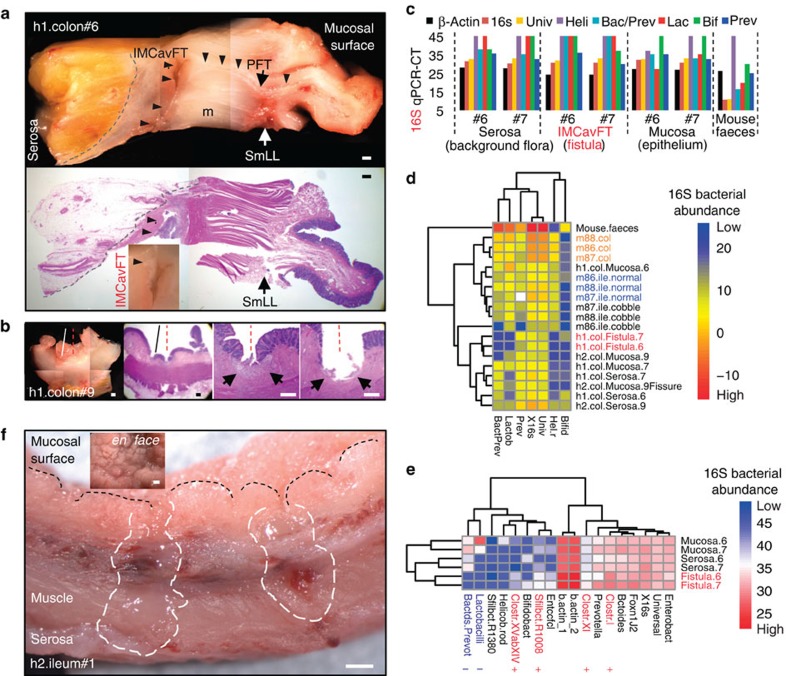
Transmural SM profiling of Crohn's disease human intestinal specimens. (**a**) Transmural surgically resected formalin-fixed (TS-SRFF) sample from colon area deemed uninvolved. SM revealed mesenteric fat/fibrosis, submucosal SM-liquefaction lesion (SmLL), ‘hairline' penetrating fistulous tracts (PFT) underneath unaffected mucosa, and complex intramural *cavernous* fistulous tract (IM-CavFT) in deep muscle layers (Supplementary Table 1 and Supplementary Figs 16–19). Histology confirmed inflammation and microscopic purulent material (inset); notice histological tissue distortion. Photo assembled from SM-images. (**b**) TS-SRFF colon sample from CD-involved area, same patient. SM revealed inter-cobblestone fissures (lines). Manual SM-micro-dissection (SMmD) of submucosa fissure lesion (dashed line). (**c**) DNA qPCR quantification of 16S bacterial families in SMmD tissues from colon of **a**. Two sample sets from the same patient (#6–7; 2 cm apart). (**d**) Cluster analysis (unsupervised, Euclidean) of 16S qPCR-CT values after subtraction (ΔCT) from β-actin for five bacterial families and two universal primers indicated IM-CavFT has distinct flora. Data from **a** and **c** and three SAMP tissues (ileum normal, cobblestone; distal colon) indicate SMmD enables SM-lesion-associated flora differentiation. (**e**) Cluster analysis of 16S qPCR-CT data of colon in **a** expanded to 13 bacterial families, with duplicated β-actin, and host *foxN1* confirms unique flora in IM-CavFT (increased segmented filamentous bacteria Sfibct.1008/clostridia). β-Actin indicates host-cell density. Serosa has distinct flora serving as an internal control to validate enriched/suppressed microbial species and determine contamination occurring during surgical–pathological handling (*in vivo* healthy serosa should be free of microbial contaminants). (**f**) TS-SRFF ileum sample from another patient. Notice transmural SmLL glossy lesions originating in inter-cobblestone (dashed line) regions. Scale bar, 1 mm.

SM was used in the early 2000s to validate the Kudo's pit pattern classification for colorectal neoplasms^41^, but no reports exist to assess 3D-structure inflammation patterns transmurally. Illustrating the importance in humans, SM was used to examine *en face* the mucosal surfaces, of colon/ileum surgical specimens from three patients with regional CD (Online Mendelian Inheritance in Man accession code 266600; http://www.omim.org). The samples (paired within-patient as affected–nonaffected) were also examined transmurally on 2–5-mm serial sections to evaluate the value of SM in examining parenchymatous specimens. As in mice, SM and histology were locally in agreement, enabling the examination of 3D abnormalities, the identification of novel types of structural lesions and their microscopic harvest in real time, with precision comparable to that of laser capture micro-dissection^42^ (Fig. 6a,b). Ileum cobblestone-to-normal mucosa transitions were remarkably similar to that of identified in mice; however, villous dimensions were larger. SM indicates cobblestone formation in humans and SAMP mice might follow similar concentric expansion dynamics (Supplementary Figs 16–19). Transmurally, SM allowed the discovery and microscopic sampling of complex cavernous fistulous tracts (CavFT) containing purulent material, running between muscle bundles in ‘normal' colon samples, underneath seemingly unaffected mucosa. DNA 16S microbial analysis from microdissected (mucosa–serosa-CavFT) matched samples revealed for the first time that such tracts have uniquely enriched segmented filamentous bacteria and clostridia species highlighting the potential for such flora–fistula complexes in perpetuating CD after surgery (65–88% recurrence)^43^,^44^. Other abnormal 3D patterns included lesions for which we propose the terms penetrating fistulous tracts, SM-liquefaction lesions and mucosal fissures (Fig. 6c–f and Supplementary Table 1). The presence of various types of transmural SM abnormalities clearly indicate that sole surface mucosal specimens as traditionally attempted^45^ are insufficient for gene expression and flora characterization of surgical intestinal specimens affected by complex IBDs. Of interest, segmented filamentous bacteria promote postnatal gut immune Th17 maturation^46^,^47^, but their role in fistula formation or CD remains unknown. Mucosa-target (compared with faecal) sampling is increasingly necessary^48^.

## Discussion

SM has been previously described in GI studies (Supplementary Fig. 1); however, applications have been traditionally limited to tissue biopsies with suboptimal spatial organ contextualization. Using SM to comprehensively assess the microscopic 3D-pattern structures of inflammation as here proposed is novel as no comparable methodologies are currently available. Most of our understanding on macroscopic intestinal disease phenotypes has arisen primarily from macroscopic assessment of lesions by trained pathologists, combined with histological characterization of inflammation severity using thin 2D sections, largely based on inflammatory cell counts and analysis of very small amounts of tissue. Herein, we propose to use a microscopic approach to comprehensively examine the integrity of entire intestinal tract segments, and characterize the disease biology, based on 3D-structural patterns as we discovered that mouse IBD stereophenotypes diverge according to genetic background (SAMP vs TNFARE). The strengths of this technique outweigh the virtually absent limitations, which are limited to additional time required for the scoring system and the learning curve that is required for implementation and SM microdissection, which can be overcome implementing defined protocols^30^.

Stereoscopic vision is fundamental in structural biology for the understanding of crystal structures^49^, and tutorials to develop vision skills to recreate 3D illusions from 2D images are readily available online. From a medical perspective, SM allows the visualization of microscopic surfaces magnified and dilated in all dimensions giving the observer an accurate sense of its real 3D topographical appearance^50^. Such perception allows observers assess and precisely manipulate most specimens without the aid of specialized devices. Although micromanipulators are available and needed to handle SM-magnified structures as small as single oocytes, our study focused on examining intestinal tissues at the villous level (villous width: 125–150 μm), and therefore was conducted harvesting tissues (>5 mg) by hand with the aid of scalpels or >26G needles as cutting/handling instruments to emphasize its potential for rapid implementation. In the early 1800s, stereoscopic images were famous as they recreated depth maps in people's minds as optical photographic illusions. Stereoscopic vision was recreated when paired pictures simultaneously taken with two-lens cameras (separated by a distance equal to the distance between human eyes) were seen simultaneously within a certain distance. Stereomicroscopes were later invented by improving separate magnifying optical pathways intended as telescopic extension of our eyes. Currently, 3D-movie industries rely on this innate virtue of the brain to reconstruct 3D structures in our minds (http://stereoworld.org/). Stereoscopy is also valuable in other difficult-to-study fields to better understand solar physics and forecasting. The National Aeronautics and Space Administration (NASA-STEREO), for instance, is studying the sun in 3D using stereoscopic data from side-viewing perspectives from two observatories; one ahead and one behind the Earth's orbit (www.nasa.gov).

In contrast to sophisticated cellular and subcellular microscopic technologies, here we propose the use of SM, which provides live stereoscopic visualization (no training needed) as a routine complementary diagnostic tool to histology and endoscopy for the rapid assessment of the 3D-structure topography of the GI tract at the villous level. With resolution comparable to low-power SEM, SM allowed us to discover novel 3D-pathological features in mouse and human IBD. Because villous abnormalities have long been observed histologically in GI pathologies (often assumed to occur at random)^21^, the thorough examination and miniaturized sampling of the mucosal topography with SM is advantageous. Applied to inbreed strains of mice, SM allowed the construction of a catalogue of 3D-SM gut lesions, and facilitated the harvest of fresh SM lesions for various assays. We identified β-actin and SDHA as stable reference genes for targeted gene expression analysis, a novel biochemically MPO-weighted exact-scoring approach, and different mouse subpopulations with bimodal morphological and functional disease severity.

Our principles for systematic SM assessment of entire intestinal surfaces are novel. In human medicine, SM was used in the 1960s to examine villous morphology ‘patchiness' on small intestinal biopsies (<3 mm) for the rapid diagnosis of coeliac disease^21^, but it remains scarcely used in clinical and research practice. Focused on a new application of SM as a research tool in IBD, we documented that SM minimizes subject and sample variability, a major statistical limitation for assessment of inflammation in mouse models of IBD.

Real-time 3D-morphology driven clinical diagnosis is becoming increasingly relevant^51^. In human medicine, the use of bedside SM of renal biopsies led to a rapid diagnosis of Fabry's disease^52^. During surgery, SM allows the selection of parathyroidectomy specimens with appropriate morphology for autotransplantation in the treatment of renal hyperparathyroidism^53^. SM has also been used to examine whole cells to visualize changes in cytoskeletal components, changes on external cell surface morphology to follow virus propagation, maturation and release from host-cell systems, and endocytosis involving selective uptake clathrin-coated vesicle mechanisms^54^. As with *in vitro* fertilization of single oocytes, SM is a promising technique for mucosal immunology and genetics due to the low cost and comparable ability to sample tissues for analysis with a range similar to laser capture micro dissection, and × 50–380 magnification SEM, but with only minimal complexity. Since many 2D-histological changes in the mucosa of human IBDs are nonspecific (shared by various diseases), it is important to consider examining intestinal disease with 3D rapid approaches. Perhaps the most relevant finding in the present study was the identification of CavFTs with intramural pathogenic (dysbiotic) gut flora in human CD, which has tremendous translational potential.

Our SM findings indicate that intestinal inflammation has unique 3D-structure pattern arrangements that replicate within the gut, clustering within specific mouse genetic lines. Specifically, we discovered cobblestone formation (SAMP 3D-stereoenterotype; flora-independent) only in wild-type SAMP mice (SPF and germ-free), SAMP strains with homozygous NOD2 mutations and in consomic SAMP with single B6-derived chromosomal replacements X, 6, 8 and 9. Comparatively, we discovered that the 3D-structure pattern of villous mini-aggregation in ileitis-prone TNF^ARE^ mice was also unique but lacked cobblestone formation (TNF^ARE^ 3D-stereoenterotype; TNF-driven). Remarkably, SM correctly predicted based on 3D-stereoenterotype whether a mouse ileum belonged either to SAMP, TNF^ARE^ or a control strain in ∼100% of cases. Of pathophysiological significance, SM also revealed that mice may have concurrent focal pathologies. SM will improve our understanding of human IBD by facilitating SM-target analysis of intestinal specimens from animals and IBD patients, which is critical for intestinal phenotyping of genetically diverse mouse and human populations and for preclinical drug testing.

## Methods

### Search of SM terms in published literature

To verify the potential applicability of SM as a routine diagnostic tool in GI research, we conducted a review of terms among 3.3 million peer-reviewed studies published in mice in Google Scholar and PubMed to compare the frequency of use for SM compared with histology, and scanning electron microscopy.

### Animals for SM catalogue and 3D-SMAP*gut* validation experiments

Mice examined in this study (30-week-old, >700 mice of both sexes) and their breeding sources are listed in Table 1. Unless specified, all mice were maintained under specific pathogen free (SPF) conditions on 12-h light–dark cycles, and fed *ad libitum* irradiated standard laboratory chow (5P00—Prolab RMH 3000; porcine animal-derived fat preserved with BHA; 6.8% content by acid hydrolysis). Corn cob and fine pine shavings were used as bedding material, which was determined not to influence the 3D-stereoenterotype of ileitis in SAMP or TNF^ARE^. When indicated, some mouse strains have paired colonies concurrently housed in separate complexes (facility A and B). Other mice were specifically purchased from approved vendors and housed until reaching 30 weeks of age in Facility A. All the procedures were approved by Case Western Reserve University's Institutional Animal Care and Use Committee and Association for Assessment and Accreditation of Laboratory Animal Care guidelines. The catalogue of SM lesions described was created after examining mice that were part of various (spontaneous and inducible) models of intestinal inflammation available in our laboratory. The goal of including various models was to allow the identification of lesion patterns that could be accounted for in the SM scoring system (3D-SMAP*gut*), which was formally tested as described in the manuscript using B6, TNF^ARE^, AKR and SAMP mice. In this study: spontaneous indicates when disease appears naturally as animals age irrespective of genetic background; and inducible disease results within a predicted period of time following the administration of an agent deemed necessary to trigger inflammation. In all the cases, animals of both sexes were used in each of the experiments presented.

### Spontaneous ileitis: B6-TNF^ARE^ and SAMP/YitFc mice

The role of TNF in the pathogenesis of Crohn's disease has long been established in humans^55^. B6 mice with the TNF^deltaARE/+^ mutation, a recognized model for TNF overproduction, were generated and PCR-genotyped as described^25^,^56^. Because homozygous animals have severe clinical signs of TNF-alpha driven inflammatory disease and short lifespans, heterozygous animals aged to 30 weeks were used. SAMP/YitFc mice have long been known to develop spontaneous disease for which some microsatellite^57^ and genetic traits have been identified^24^,^58^. A whole-genome and transcriptome sequencing project to enable the elucidation of (epi)genetic factors in SAMP gut disease has been launched at CWRU in collaboration with the Beijing Genomics Institute 59.

### Spontaneous colitis: SAMP6 and in FVB/NJ ^mdra1−/−^ mice

SAMP6 mice originally obtained from Harlan Laboratories Inc. have been maintained by FC at CWRU. SAMP6 mice are derived from AKR/J as SAMP1 mice by selection and possible genetic outcross contamination. SAMP6 belong to the senescence-accelerated mouse prone phenotype and have susceptibility to osteoporosis/osteopenia, and the occurrence of colitis^60^. FVB/NJ mice initially engineered to carry a homozygous mutation in drug transporter^61^ *mdra1*^−/−^ are known to have heavier ileum with respect to other mouse strains, and are prone to develop colitis^62^. To determine the 3D-stereoenterotype of the latter mice, adult mice were purchased as retired breeders from Taconic.

### Faecal flora homogenization

Before the commencement of any relevant comparative experiment in the present study, animals were subjected either to a period of temporary co-housing in which young animals from intended treatment groups or separately maintained colonies were co-housed at the time of weaning, and treatments given at 30 weeks of age, or animals were orally gavaged a composite of fresh and dry faecal matter^30^.

### Inducible colitis

To induce DSS colitis^36^, mice were offered filtered–sterilized 3% (w/v) DSS as their sole drinking water for 7 days *ad libitum*, then mice were euthanized for tissue harvest. To minimize cage-to-cage clustered variability in inducible experiments, animals were housed individually and exposed to a composite mixture of faecal matter (slurry; sterile water mixed with soiled bedding material from all experimental cages with excrements accumulated 7 days before the experiment, and gavaged 300 μl of faecal suspensions). Between 100–200 g of composite faeces were added to fresh bedding in each cage. After 3 days of exposure (that is pre-experimental fecal homogenization period; PreFeH^30^), homogenization period), experiments started. Irradiated autoclaved food was provided in all the cases. Bouin's fixed intestinal specimens from animals from AOM/DSS, and T-cell transfer colitis in SCID mice, available from concurrent experiments in our laboratory^63^ were also examined.

### *Salmonella* and *Clostridium difficile* murine models of infection

Mice infected with either *Salmonella* or *Clostridium difficile* were also examined. A pre-treatment streptomycin method was used for acute *Salmonella* infection colitis in which water and food were withdrawn 4 h before the oral administration of 20 mg of streptomycin^64^. Twenty-four hours later, 5-week-old AKR and SAMP mice were gavaged 10^9^ CFU of streptomycin-resistant *Salmonella enterica* serovar Typhimurium strain SL1344^65^ per mouse, and then euthanized 48 h after infection. Acute *C. difficile* infection of 7-, 30- and 50-week-old SAMP, B6 and AKR mice was conducted using bacterial strain 630 (10^9^ CFU per mouse) 24 h after 4 days of oral clindamycin in the drinking water^66^ and one intraperitoneal dose 24 h before infection. Acute *C. difficile* infections were also studied in SAMP mice under GF conditions and 3 days post infection, without the need of clindamycin. SAMP GF mice are maintained at CWRU. Bacterial colonization was verified by identifying increased numbers of CFU after plating appropriate faecal dilutions on MacConkey agar supplemented with streptomycin (50 μg ml^−1^) for Salmonella^64^, or *C. difficile* fructose agar (Oxoid, CM0601) supplemented with D-cycloserine and cefoxitin (250 and 8 μg ml^−1^; Oxoid, SR0096) for *C. difficile*^67^.

### Endoscopy

Colonoscopy and ileoscopy was performed under general anaesthesia with inhaled isofluorane and using a flexible digital human ureteroscope as recently described in great visual step-by-step detail^26^. In brief, mice were anaesthetised by administering 4–5% of isoflurane in 100% oxygen at a rate of 0.2–0.5 l min^−1^, and use 0.5–2% for maintenance. Following immobilization of the anaesthetised mouse, the ureteroscope was inserted rectally while video recording the endoscopic evaluation. A decimal-integrated colonic inflammation-and-cancer-validated scoring system was used. A form we have created with novel integration of suboptimal integer scoring scales was used after the examination of the video images. For ileoscopy, a surgical and postmortem approach was used^26^.

### Tissue harvest and fixation

Ilea and colon were removed from mice in all the cases to determine the presence of SM abnormalities on the mucosal surface. Caecum was observed in some mice, which exhibited lesions comparable to the distal colon. SM and histological examination was conducted on intestinal tissues harvested from all mice. Bouin's, carnoy, neutral buffered formalin, PBS and RNA-later fixed tissues were tested as fixative, moisturizing or nuclei acid stabilizers prior conducting SM assessment to determine suitability. In brief, fresh intestinal specimens were placed on sterile petri dishes (and a black background), dry or moistened with cold PBS, for rapid SM-dissection of tissues for functional analysis (for example, RNA, TEER) and then submerged in fixative overnight, later replaced with 70% ethanol for preservation until SM and histology. Bouin's fixative was selected as preferred for SM profiling; however, all tested reagents were appropriate for analysis despite the semi-transparent appearance of tissues. Bouin's fixative was the only coloured solution tested; it is yellow and confers the tissues a yellowish tonality when freshly examined under SM, or dark brownish after a few weeks of storage. 3D-SMAP*gut* was possible with all fixatives, regardless of coloration by experienced observers.

### Autolysis and decomposition post fixation

To determine the effect of natural autolysis of the intestinal mucosa on the SM appearance of the mucosa in ileum and colon following death, 30-week-old SPF-SAMP mice (*n*=8) were euthanized, left at room temperature and then processed for harvest of tissues at one mouse per hour intervals. SM and histology were conducted as for other experiments. When eroded villi were first observed, experiments were conducted to determine whether villous erosion resulted from bacterial decomposition in ethanol-preserved Bouin's-fixed specimens stored at room temperature. In brief, specimens examined fresh, during fixation and at 2 and 60 days after fixation, showed that erosion was not due to or worsened with decomposition.

### Mouse histological inflammation scores and SM validation

Histological evaluation of inflammation severity for SM correlation analysis was determined in haematoxylin and eosin-stained 5-μm-thick sections using a semi-quantitative scoring system^64^. Colon scores were given for percentage of re-epithelialization (0–4), ulceration (0–3), active (0–12) and chronic inflammation (0–12), and transmural inflammation (0–12). For ileum, scores were given for the severity (0–4) for five indices (0–3, Supplementary Fig. 9). Total scores resulted from adding the individual index scores. One certified human pathologist determined all scores blindly. The catalogue of 3D-SM abnormalities created was characterized by examining the cellular structural pattern via histological examination of SM-dissected tissues in consultation with an additional pathologist, and SEM analysis.

### Scanning electron microscopy

SEM analysis was conducted on Bouin's-fixed 70%-ethanol-preserved tissues after SM examination/dissection on 16 samples representing all SM-lesion category types in at least duplicate or triplicate. Before SEM analysis (5–7 days), samples were re-fixed by immersion in triple aldehyde-DMSO mixture for 3 h at room temperature, then washed (three times, 5 min each) with 0.1 M HEPES buffer (pH 7.3), then post-fixed in 0.1% osmium tetraoxide solution (3 h, 23 °C, in the dark) and finally washed with distilled water (5 times, 5 min each). Tissue dehydration was accomplished by serial passages through aqueous ethanol:water solutions at 50, 70, 90, (30 min, 1 × ) and 100% (30 min, 2 × ) with a final chemical dehydration step with hexamethyldisilazane (HMDS; 100% ethanol: HMDS, 1:1, 2 × , 10 min; followed by 100% HMDS 3 × until sample was dry at room temperature). Samples were sputtered with a 10 nm layer of gold and examined at 15 kV with JEOL scanning microscope. Compared with SM, tissues suffered minimal dehydration artifacts, but gold sputtering prevented SEM images from providing information about structures covered by mucus/fibrin.

### SM and decimal scoring system

We repeatedly evaluated the technical and mathematical suitability of various scoring systems and after nine revisited drafts, we developed a final version of collective parameters, not previously reported to describe the 3D appearance of the intestinal mucosa. Supplementary Fig. 8 has the form and criteria used to assess the last part (10 cm) of the mouse ileum and the entire colon. The last 10 cm of ileum corresponded to 1/3 of the small intestinal length and the area where lesions were identified in all cases if any areas of ileitis were present^30^. Ten centimetres of tissue were deemed ideal for standardized scoring because most healthy colon's length is ∼10 cm (colitis almost always affect the last 5–6 cm), and because in the SAMP and TNF^ARE^ ileitis models examined at 30 weeks of age consistently have lesions in the distal 10 cm of the small intestine. Notice, however, that SAMP enteritis is not restricted to the ileum (Supplementary Fig. 3), and TNF^ARE^ enteritis has a gradual transition in the most severe cases that range severe by the ileo-cecal junction to mild/normal morphology by 6–8 cm before the caecum (Supplementary Fig. 10).

### MPO activity and data re-scaling for SM comparability

Tissues obtained by SM-dissection were tested for MPO activity^31^ as described^64^, with important modifications validated for this study in the acquisition of data and interpretation of time series spectrometry changes in absorbance over 5 min of incubation at 28 °C using 96-well plate formats^30^. Instead of inferring the data from only three time point measurements as commonly reported, which had a great sample-to-sample variability due to incubation or loading-of-reagent artifacts, we automatically recorded the OD changes every 30 s to create a kinetic curve from which the steepest slope was identified to extract slope information needed to make precise quantifications^30^. To incorporate the MPO activity into the SM scoring system, we discovered that the most effective method would require the Log_2_ transformation of the data and subsequent re-scaling to ensure a more linear distribution of the MPO estimates and improve the discriminatory power of MPO for samples with low enzymatic index. Re-scaling was conducted for each value using the equation: *y*=minnew + (*x*−minold) × (maxnew−minnew)/(maxold−minold), where *y* is the new (re-scaled) value; *x* is the value to be re-scaled, minold and maxold are the minimum and maximum values that encompass the (old) data set to be re-scaled, and minew and maxnew are the low and high values to be set as minimum and maximum limits for the (new) re-scaled data; that is, between 1 and 2 for the lowest and highest MPO values, respectively, in the entire experiment or laboratory. Notice that MPO activity is best if determined with fresh tissues or after 24 h after storage at −80 °C (the preferred method for large experimental groups, used in this study). If the technical conditions cannot be reproduced (fresh tissues and timing), re-scale the MPO data and interpret findings in the context of the experimental set alone. The ability of MPO to differentiate groups is lost as tissues lose MPO activity over time.

### Transepithelial electrical resistance (TEER)

TEER assays were performed as described^34^. In brief, SM-dissected 3 × 3 mm tissue samples rinsed with 0.9% NaCl were placed with the apical side up on separate precut 0.4-μm pore size membranes (Costar Incorporated, Corning, NY). The tissue-membrane mounts were ‘sandwich' placed between two custom-made Plexiglass discs with laser-cut holes to create a 2-mm diameter circumference of tissue membrane for exposure to TEER electric currents. ‘Sandwich' mounts were placed into Snapwell inserts (Costar) apical side up, and fitted into a six-well plate. SM was used to verify the tissue was properly placed. RPMI media were added on both sides of the mounts (2 ml basally and 500 μl apically) to ensure mounts were surrounded by the liquid media. TEER readings were taken using an EVOM voltmeter with an EndOhm chamber attachment (World Precision Instruments, FL) immediately after assembling transwell apparati and after 1 and 2 h of incubation at 37 °C.

### RNA and real-time qPCR

Following physical tissue homogenization (with ∼200 mg of 1.4-mm ceramic beads, 4,000 r.p.m.), total RNA was isolated from homogenates using a commercial kit (Qiagen RNeasy Mini, 74106). Relative mRNA expression was quantified using a Roche SYBR Green platform. For each PCR assay, a master mix was prepared for each gene of interest and the housekeeping genes using FastStart Universal SYBR Green Master (Roche, 04913914001), Gibco UltraPure Distilled DNase RNase free water (Life Technologies, 10977-015) and 10 μM of each forward and reverse primers (Integrated DNA Technologies, Supplementary Table 2). To ensure elimination of host DNA, samples were treated with RNase-Free DNase (Qiagen, 79254) as part of the RNA extraction protocol. RNA quantification was done spectrophotometrically (Take3 Micro-Volume Plate, Eon Microplate Spectrophotometer, BioTek,) and validated using a Qubit 2.0 Fluorometer (Life Technologies). High Capacity cDNA Reverse Transcription Kit (Applied Biosystems, 4368814) was used for stabilization of RNA as cDNA using random hexamers and 1 μg of total RNA in a final reaction volume of 20 μl. The final adjusted cDNA concentration was 2 μg ml^−1^. qPCR amplification of cDNA samples was performed using clear 96-well plates (Roche, foil sealing 05102413001) and the Roche 480 LightCycler SYBR Green run template settings (hot start 95 °C, 10 min and 40 amplification cycles; 95 °C, 15 s; 60 °C, 30 s; 72 °C, 30 s). If primers required distinct melting temperatures, it was reset accordingly. The default melt curve setting was used to examine primer performance. Samples were run as singlets in each plate to allow the inclusion of all samples from a given mouse in a group, but experiments were repeated at least twice to determine variability and reproducibility (in this study, correlation analysis of duplicate experiments, >0.88–0.98; Supplementary Fig. 11). One liver, ileum and colon samples from a 30-week-old SAMP mouse were run in every plate throughout the study to monitor and control for plate-to-plate variability. Plates with abnormal performance were examined for anomalies and repeated in duplicate for confirmation.

### Surgical specimens from humans with CD

The present study also used SM to examine the 3D-topograpgy and morphology of formalin-fixed intestinal surgical specimens once the pathological characterization of the tissues by the Department of Pathology, University Hospitals Case Medical Center was completed following approved IRB/hospital protocols for handling of biological discarded pathology materials. Informed consent was not required. Specimens were examined after deemed material for disposal by Pathology and anonymized. Handling and reporting of data related to these experiments was therefore conducted as unidentified specimens/sources also in agreement with approved research IRB protocols.

### qPCR enumeration of 16S-based microbial communities relative to β*-*actin

We quantified specific bacterial communities at the family level relative to the abundance of β-actin (host-cell gene marker) or total 16S DNA using DNA extracts from SM-dissected tissues and real-time qPCR. β-Actin was deemed appropriate for primary normalization since all nucleated host cells carry two gene copies per cell. Data were also examined and presented using total 16S for normalization. All primers used had been previously validated (Supplementary Table 2). qPCR quantification has been a sensitive method of enumeration of bacteria in fresh and 10%-formalin-fixed 70%-ethanol-preserved intestinal tissues, even at the genus level^68^,^69^. Cross contamination during SM-dissection was minimized by using new/flamed scalpel blades or needles for each sample collection. The human specimens that were available for this study were only available as formalin fixed, therefore, the whole specimens were deemed to be contaminated with background faecal flora. To determine flora differences between the mucosa and the cavernous fistulous tracts (CavFT, see term descriptions in Supplementary Table 1), paired samples for a particular transmural sample were orderly collected (fistula first, then serosa, last mucosa). DNA extraction was performed from fresh and fixed tissues using a Qiagen kit (Gentra Systems, Minneapolis, MN), following the recommendations for bacteria, and after a period of tissue digestion with proteinase K (ref. 70). Pure DNA quantification was conducted with fluorescence and Qubit and then adjusted to equimolar concentrations using PCR grade water. Gel electrophoresis and Sanger sequencing of PCR products were conducted to verify amplification quality for β-actin in selected wells using sequence BLAST alignment.

### Tissue handling for 3D-SMAPgut

Although the examination and scoring of fresh tissues is possible with the present system, we recommend to score fixed tissue, which makes samples opaque (Bouin's solution ideally). The semi-transparency of fresh tissues makes assessment/scoring more difficult and slow, which may be detrimental on specimens for downstream analysis. Immersion of sample in cold PBS or RNAlater, placing tissues on a surface with crushed ice or working on a 4 °C walk-in cooler are appropriate measures for assays where samples required fast cell stabilization, including sampling for RNA analysis. Because SM assesses the integrity of the gut surface, there are conditions that need to be avoided to allow the maximum amount of tissue to be assessed (collect miniature sample), and prevent technical errors that alter the 3D gut morphology. Avoid tissues to dry while fresh samples are SM-collected. With desiccation, the mucous layer will harden and become fixed in Bouin's as an artifact. Normal tissues have clean villi, but as a common abnormality dense muco-fibrinous layers persist during fixation. SEM indicates that such layers can be present in combination with villous abnormalities underneath (visualized only partly with SM, but not with SEM due to required metal coating of fixed samples, Supplementary Fig. 6). Intestinal samples pinned on the cork or black Styrofoam (Supplementary Movie 1) with the mucosa facing down destroy the architecture of the mucosa by deformation, abrasion or solidification of faecal matter within mucous. Avoid tissue damage with forceps or scissors as tissue is opened; however, those artifacts are easy to differentiate from focal pathologies.

### 3D-SMAP*gut* scoring rationale

As described in the manuscript, a 3D-SMAP*gut* score contains information regarding the extent of abnormal mucosa and the lesions that account for it. The scale developed for the small intestine ranges from 0.000 to 1,000.987. A score of 0 means healthy mucosa, while 456.789 indicates that the segment examined has an average of 45.6% of abnormal mucosa per linear (tubular) centimetre of gut examined, while the decimals indicate the SM-lesion categories in order of prevalence. The rationale behind this calculation strategy was determined to best address the inflammatory condition for each organ separately due to anatomical and inflammatory differences between small and large intestine. Specifically, the large colon has a smooth mucosal surface compared with the small intestine (which has villi of various lengths), has different 3D-SM inflammation structures and shortens during inflammation. To better understand the rationale, it is important to notice the type of lesion categorization that was necessary to comprehensively include all SM anomalies observed in this study (Supplementary Fig. 1–19). For small intestine, the protocol takes into consideration the various 3D arrangements that villi can take over the space instead of mucosal surface smoothness, which is absent due to the presence of villi. For the large colon, we observed that tissues exhibit various levels of corrugation in the absence of villi, which correlates with treatment, MPO activity and histological scores. The categorization of SM-lesion anomalies observed for small and large intestine in mice were derived from intense examination of ileum and colon, respectively, but it was verified they apply to duodenum and jejunum in the small intestine, and caecum in the large colon.

With respect to the materials needed, the assessment of intestinal inflammation requires a stereomicroscope and the tissues collected from experimental mice fixed for histology. A digital camera recording system is optional, but if present, allows data archival. We used Bouin's solution and 3–18 h (overnight) fixation, with replacement of solution with 70% ethanol for tissue preservation. Bouin's solution makes the tissue opaque with yellowish discoloration, which allows shadow formation when illuminated from the proximal (oral) side of the intestinal tract (Supplementary Movie 1). Incident illumination from the side is important because shadow formation is one of the most important cues our brain uses to infer 3D structures^30^.

### Step-by-step scoring protocol

*Set up the microscope*. Use these guidelines, fine-point forceps, scalpel, blades and insulin needles for tissue handling. Use a spray bottle with 70% ethanol to moisten the tissues when needed, and an indelible marker or pencil for recording of data and labelling. Paper towels, gloves and proper attire when handling fixative chemicals are also needed.

*Blind-fashion examination*. As with any other quantitative/qualitative assessment and scoring protocol, it is important to be aware of the potential of inadvertent technical misclassification bias and boredom or mental exertion if a larger number of samples are to be examined. Take measures to prevent uneven accumulation of errors across experimental groups of animals. Preventive strategies include examining samples from random animals across all groups (instead of examining all animals group per group), as examination progresses, using masking methods to prevent examiners from knowing the treatment assignment. Alternatively, use masking tape to cover the tube labels where samples are submitted with treatment assignment information and do the analysis blindly. Over time, also use reference images provided in the manuscript and create your own to serve as comparators to retrain our abilities to estimate the percentage of abnormal/normal areas more accurately. This will make estimations over time and across experiments more stable, because unlike histology, it is easy to delineate a few areas and estimate a percentage number with relative precision over time.

Discuss and compare with peers scoring estimates and use imageJ to obtain the exact measurements for comparison. Use the guidelines in the manuscript for reinforcement, and print posters or handouts for quick reference during scoring. Do not rely on memory; be aware that memorized events shift in our minds gradually and unnoticeably away from reality every time a fact is recalled. It is important to obtain ∼11 cm of tissue from the mouse for scoring. Note in the Supplementary Movie 1 that the length of a 50 ml conic tube allows the collection of such segments. Avoid creating tissue damage to the distal segment of the intestinal tract during handling; consider the use of blunt tissue forceps. When ready, take specimen out of the transportation solution (for example, ethanol 70%) and remove the excess solution by laying the specimen on a paper towel on the serosa side. Dry tissues provide the best contrast for imaging and direct scoring. Mount the tissue with the distal segment to your left and the light on the right-hand side over a flat surface: for instance, a petri dish with a black background. The light angle should be between 20° and 30° from the right.

Focus the specimen to cover a 1-cm field, and start scoring. First determine the percentage of abnormal mucosa affected with all kinds of SM-lesion categories listed in the scoring form (Supplementary Fig. 8); then the percentage of mucosa that looks normal (healthy finger-tip/tongue-like/plate-shaped/leaf-shaped villi that appears thin and with ample space around; or smooth thin surface/folds in the colon) compared with a normal IBD-free mouse control of the same age or images in this manuscript. Note that inflamed/thickened villi (blunt or polygonal villi), which are more difficult to assess, are better estimated by subtraction of abnormal+normal areas from 100%. These data will also provide an overall estimate of the general sample quality and health with respect to villi morphology. These estimations are very intuitive (see manuscript) and represent the foundation and minimum scoring data that strengths this system. Then optionally, enumerate and record the types of lesions present in each centimetre as indicated in the Supplementary Figs 8–10.

If a SM video is desirable for archival purposes, make the videos as indicated in Supplementary Movie 1. A critical aspect for scoring efficiency based on the assessment of videos is to minimize the delays that the examiner will encounter if the video is too long and not predictable. Examiners will spend more time and effort as they will have to observe the entire video clip for a particular centimetre and then decide which the best still frame is to return to it for scoring. This process is mentally demanding. The critical best practice is to zoom to a 1.05-cm-width area, then zoom in and out as desired to gain insight on local lesions if relevant, and stay still for 3–4 s before moving to the next 1.05-cm frame. Repeat this process for the next centimetre. Always start with the most distal centimetre, to the left of the specimen and video. By doing so, the video frames are predictable. Make the videos as short as possible. If too long, make two videos, one for scoring at 1.05-cm frames, the other one with more details. This approach in difficult cases (that is, more lesions to characterize) makes the scoring process more efficient. Take photographs separately in special cases. Store the data in an Excel file like the one example provided for downstream analysis and for meta-analysis purposes in the future (see template file in Supplementary Data 1).

### Training modules for implementation of 3D-SMAP

To enable the implementation of SM-based scoring protocols (that is, 3D-SMAP*gut*), as well as and the use of SM in routine GI experimentation, we prepared the actual training (Microsoft power point) modules used in this manuscript based on Supplementary Figs 1–15. For testing the scoring ability of trainees, these modules were tailored to the need of the trainees and type of SM-lesion difficulty and research interest by adding test images obtained from relevant case specimens^30^.

### VideoSM settings for ImageJ analysis of villi

This paper was designed to enable the use of ImageJ, a free public-domain software developed by the National Institutes of Health for image processing and analysis. In short, fixed tissues were placed on anti-glare dark background and millimeter ruler used as reference for image taking. Colour and monochrome Tagged Image File Format (TIFF) images without compression were taken using (AmScope-MT Software MA1000; resolution 3,664 × 2,748) with ∼30° angle lateral/incident illumination provided by dual LED sources, independently flexible (3+3 Watts; 3,200K colour temperature). With the distal end of intestinal specimen to the observer's left, illumination produced the best 3D data in proximal-to-distal direction, that is, right-hand to left-hand orientation. Brightness, exposure time and light intensity were manually adjusted to have the clearest image possible. Magnification level was set at the discretion of the operator, but, in case of multiple tissue comparisons, the same magnification was used. For standardized video SM, we recommend to zoom to cover 1.1 cm field to enable the proper examination of tissues cm by cm (Supplementary Movie 1).

### ImageJ analysis

Image J is a public-domain Java image processing programme downloadable for Windows, Mac and Linux developed at the NIH (http://imagej.nih.gov). Features such as calculation of area and pixel values by user-define lines or by drawing regions of interest (ROIs), density histograms, line profile plots, angles, distance and several other complex plugins, make ImageJ an excellent tool for studying SM images. Black/White TIFF image were analysed using ImageJ v1.48r for Windows. First, a known length line was drawn between two reference points over the ruler photographed with the intestinal tissue. Longer lines provided greater accuracy. The image scale was set using the ‘Set Scale' tool (Analyze >Set Scale). Villi count and operator naked-eye validation was done using Plot Profile and ROI manager instruments. Several longitudinal lines were drawn haphazardly and stored in ROI Manager in Analyze >Tools >ROI manager, then Plot Profile analysis was performed by consecutively selection of lines using tool available in Analyze >Plot Profile. Each produced histogram can be exported for statistical analysis using the List button. Each histogram (for example, line plots if binned to pixel or coordinate scales) are a representation of the grayscale intensity intercept by the line and for our purpose, can be summarized as the dark is the ‘bottom' of the crypt (black) and the light is the apical part of the villus (white, shiny reflective convexity of villi). Following this criteria, it was possible to define in the histogram, the space between Blacks and Blacks (BB intervals) as a representation of the space between villi. In this way, the number of villi intercepted by the drawn line was calculated and validated manually. Difference in villi count was conducted using the same method by longitudinal, transversal and random lines. To determine whether the count of villi could change using different line direction and angulation, the same analysis was carried out using longitudinal, transversal and random line. Line plot analysis was found to be a reliable and sensible method also in images of tissues affected by other focal lesions. Smoothing of images was examined, but it was deemed detrimental if smoothing of >2 pixels was chosen for villi line plot enumeration.

For the analysis of discrete lesion areas (for example, percentage of abnormal mucosa), the built-in tools within ImageJ were also used. For the training session where observers were presented with SM images delineated with the area edges, imported and scaled (photo or stitched video SM images) photo files were used to draw line polygons around areas of interest (for example, cobblestone lesions) defined by consensus between A.R.-P. and D.P. In some cases, polygons were drawn at random for training purposes. To enable comparability and scalability, the scale units were set in millimetres. Area measures relevant to SM are automatically generated by the Analysis tool in ImageJ. The bubble plots presented in Supplementary Fig. 4 were created in SPSS using area data (mm^2^) and the associated ImageJ *xy* coordinates for corresponding area centroids. The exact percentage of abnormal mucosa was determined by comparing the total sum of selected areas to the total gut imaged referent polygon area (edges of gut surface in each photograph). Other ImageJ-based phenotypic analysis using segmentation algorithms have been successfully conducted and are a part of ongoing automation efforts of SM-video image analysis (data not shown). See software/examples documentation at http://imagej.nih.gov/ij/.

### Statistics

To comply with required reporting standards, this methodological paper intended to examine a large number of animals from various strains and treatments to ensure a catalogue of lesions representing natural and induced forms of inflammation could be represented in the scoring form developed. In most cases, sample sizes were based on published studies, >3 per group; with experiments repeated at least once, and a mode of six mice per group. As such, no intestinal tissues were excluded from the SM study; improperly preserved samples were also examined to characterize the SM appearance during autolysis. When needed, STATA was used to determine sample size effects. Randomization of animals was conducted in all the experiments a priori based on a list of experimental animal available. Blinding was maintained in all stages via the use of non-informative codes, which was revealed once the analysis was completed or images were in preparation. Parametric statistics were used when data fulfilled test criteria. Log-transformation was conducted for MPO analysis as described to improve normalization. A *P* value was significant if it was <0.05. Nonparametric statistics were used as alternative. All inferential statistics were conducted using R, SPPS or STATA software.

## Additional information

**How to cite this article:** Rodriguez-Palacios, A. *et al.* Stereomicroscopic 3D-pattern profiling of murine and human intestinal inflammation reveals unique structural phenotypes. *Nat. Commun.* 6:7577 doi: 10.1038/ncomms8577 (2015).

## Supplementary Material

Supplementary Figures, Tables and References Supplementary Figures 1-19, Supplementary Tables 1-2 and Supplementary References

Supplementary Movie 1 Stereomicroscopy video preparation for 3D-structure pattern profiling of murine intestinal inflammation. In this video the authors describe the step procedures from the collection of intestinal specimens in mice to the settings needed to record a SM-video, and provide examples.

Supplementary Data 1 Spreadsheet template for storage of 3D-SAMPgut data. This excel file is a template example of how to enter and store data for ulterior analysis (multivariable, multivariate, and meta-analysis) and plotting purposes.

## Figures and Tables

**Table 1:** Mouse strains/models of IBD examined in this study.

**Strain**	**Breeding source**	**Reported digestive disease phenotype**	**Main reported and studied GI disease phenotypes**	**SM Cobblestones**	**Other SM lesions**
A/J	Jackson	No*		No	Yes
AKR/J	Jackson, CWRU	No*		No	Yes
BALB/C	Jackson	No*		No	Yes
CBA/J	Jackson	No*		No	Yes
C3H/HEJ	Jackson	No*		No	Yes
C57BL6/J	Jackson	No*		No	Yes
C57BL6/NJ	CWRU	No*		No	Yes
C57BL6/NJ^redfoxp3^	Jackson	No*		No	Yes
DBA/2J	Jackson	No*		No	Yes
SCID	CWRU	No*		No	Yes
FVB/NJ	Jackson	No*		No	Yes
FVB/129P2^mdr1a−/−^	Taconic	Yes, spontaneous	Colitis	No	Yes
C57BL/6J^TNFΔARE/+^	CWRU	Yes, spontaneous	Ileitis, arthritis	No	Yes
C57BL/6J.129S^TNFΔARE/+^	CWRU	Yes, spontaneous	Ileitis, arthritis	No	Yes
SAMP1	CWRU	Yes, spontaneous	Ileitis,	Yes	Yes
SAMP1/YitFc	CWRU	Yes, spontaneous	Ileitis	Yes	Yes
SAMP1/YitFc ^NOD2−/−^	CWRU	Yes, spontaneous	Ileitis	Yes	Yes
SAMP1/YitFc—Germ-free	CWRU/Taconic	Yes, spontaneous	Ileitis	Yes	Yes
Consomic SAMP with single replacement of C57BL6/J-derived of chromosomes X, 6, 8, and 9	CWRU	Yes, spontaneous	Ileitis	Yes	Yes
SAMP6	Harlan, CWRU	Yes, spontaneous	Colitis	No	Yes
DSS-induced	CWRU	Inducible	Colitis	—	Yes
AOM/DSS induced	CWRU	Inducible	Colitis	—	Yes
RBhigh into SCID induced	CWRU	Inducible	Colitis	—	Yes

^*^Strains in the top third of the list were selected from strains for which the genome has been sequenced and that are phenotypically well characterized (www.sanger.ac.uk/resources/mouse/genomes/). Strains that are not referenced in the manuscript are part of ongoing genetic projects in our laboratory.

**Table 2:** Gene expression stability of candidate human housekeeping genes indicates β-actin is suitable for SM-target within-animal paired sampling and DGE analysis in mice.

**CT values, mean±s.d.**	**β-actin**	**SDHA**	**18S**
*By organ*			
Caecum	16.267±1.387	20.273±1.476	11.482±3.184
Ileum	16.265±1.686	20.792±1.735	12.178±3.457
Family pool s.d.	**1.514**	**1.606**	**3.317**
Mean sum sq.	**2.29**	**2.58**	**11.0**
ANOVA F	F_1,245_=<0.01	F_1,233_=6.22	F_1,246_=2.7
*P* value	0.992^NS^	0.013^NS^	0.101^NS^
			
*By treatment*			
PBS control	16.421±1.682*	20.717±1.834*	12.107±3.629*
Dexamethasone	16.338±1.455*	20.690±1.322*	12.581±3.073*
3% DSS	15.619±0.720^†^	19.731±0.947^†^	9.934±1.373^†^
Family pool s.d.	**1.491**	**1.589**	**3.189**
Mean sum sq.	**2.22**	**2.53**	**10.2**
ANOVA F	F_2,245_=5.53	F_2,233_=7.46	F_2,246_=10.69
*P* value	0.004**	0.001**	<0.001***
			
*By mouse strain*			
C57BL/6	15.413±0.778*	19.395±0.767*	8.931±0.672*
SAMP1/YitFc	16.200±1.478^†^	20.596±1.545^†^	11.771±2.742^†^
AKR/J	17.356±1.688^‡^	21.428±2.118^‡^	14.859±4.591^‡^
Family pool s.d.	**1.423**	**1.535**	**2.887**
Mean sum sq.	**2.03**	**2.36**	**8.33**
ANOVA F	F_2,245_=17.89	F_2,233_=16.35	F_2,245_=39.95
*P* value	0.004**	<0.001***	<0.0001**

NS, not significant.

Analyses of variance (ANOVAs) parameters. β-Actin and *SDHA* are comparably the most stable of the three genes tested, while 18S is the most variable/uncertain (notice its extreme family pool s.d. and mean sum squares). See also Supplementary Fig. 11. Analysis of arithmetic values combining β-actin and *SDHA* (CT average, or ratio) indicates that SDHA could be better used as a secondary housekeeping gene to independently verify DGE profile findings derived from β-actin (gene network reproducibility), instead of combining them into a single averaged value as reference. Bold font highlights variability parameters; note 18S variation doubles that of B-actin and SDHA.
