# Supplementary material for: Stereomicroscopic 3D-pattern profiling of murine and human intestinal inflammation reveals unique structural phenotypes
Source: Nat Commun. 2015 Jul 8;6:7577. doi: 10.1038/ncomms8577 (PMC4510646; doi:10.1038/ncomms8577)
Supplement: Supplementary Figures, Tables and References — Supplementary Figures 1-19, Supplementary Tables 1-2 and Supplementary References [file ncomms8577-s1.pdf]

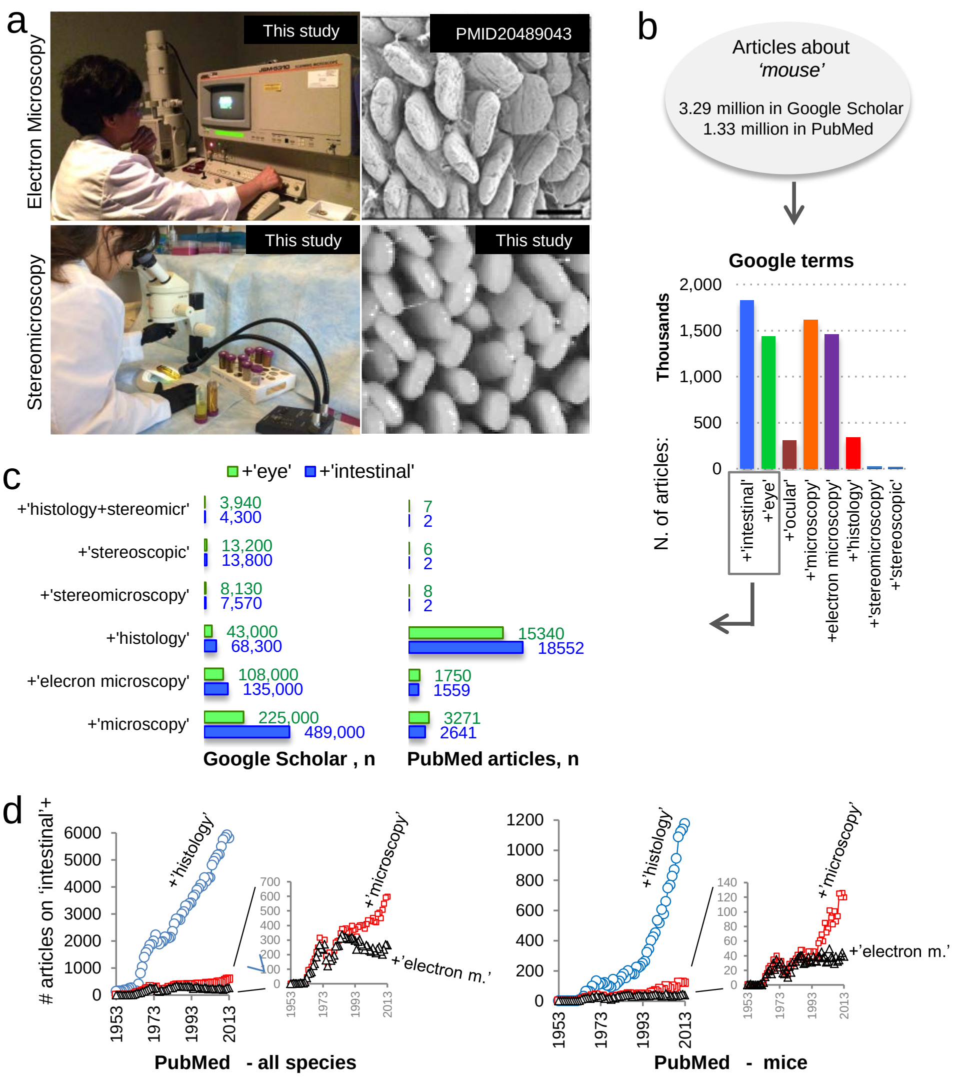

Supplementary Figure 1. Stereomicroscopy (SM) and scanning electron microscopy (SEM) in gastrointestinal (GI) research. (**a**) SM produces real-time images comparable to low-power SEM. That resolution showed to be effective to evaluate the intestinal 3D-topography, rapidly, over entire intestinal specimens. Notice image from a study[1] (PMID20489043) where technical-intensive SEM was used to differentiate intestine villi of 129/SVJ wild type from knockout mice (Permission obtained from The American Physiological Society, Nighot P.K. & Blikslager A.T., Am. J. Physiol. Gastrointest. Liver Physiol. 299(2): G449-456; 2010). (**b**) A search of key terms indicated that SM has been scarcely used in published GI studies using mice with respect to other key terms; August 08, 2014. The number of studies was derived from a selection of relevant terms (in quotation marks) using Google Scholar and Pubmed. Bar chart, articles for 'mouse' + 'other terms' document best terms for hierarchical search. (**c**) Hierarchical search (mouse + organ + diagnostic tool); notice scarcity of SM studies. (**d**) Rapid growth of publications citing histology since the mid-1980s with respect to studies citing Electron M. SM has the potential to be a ubiquitous complementary diagnostic tool to histology, adding 3D-data to routine GI research.

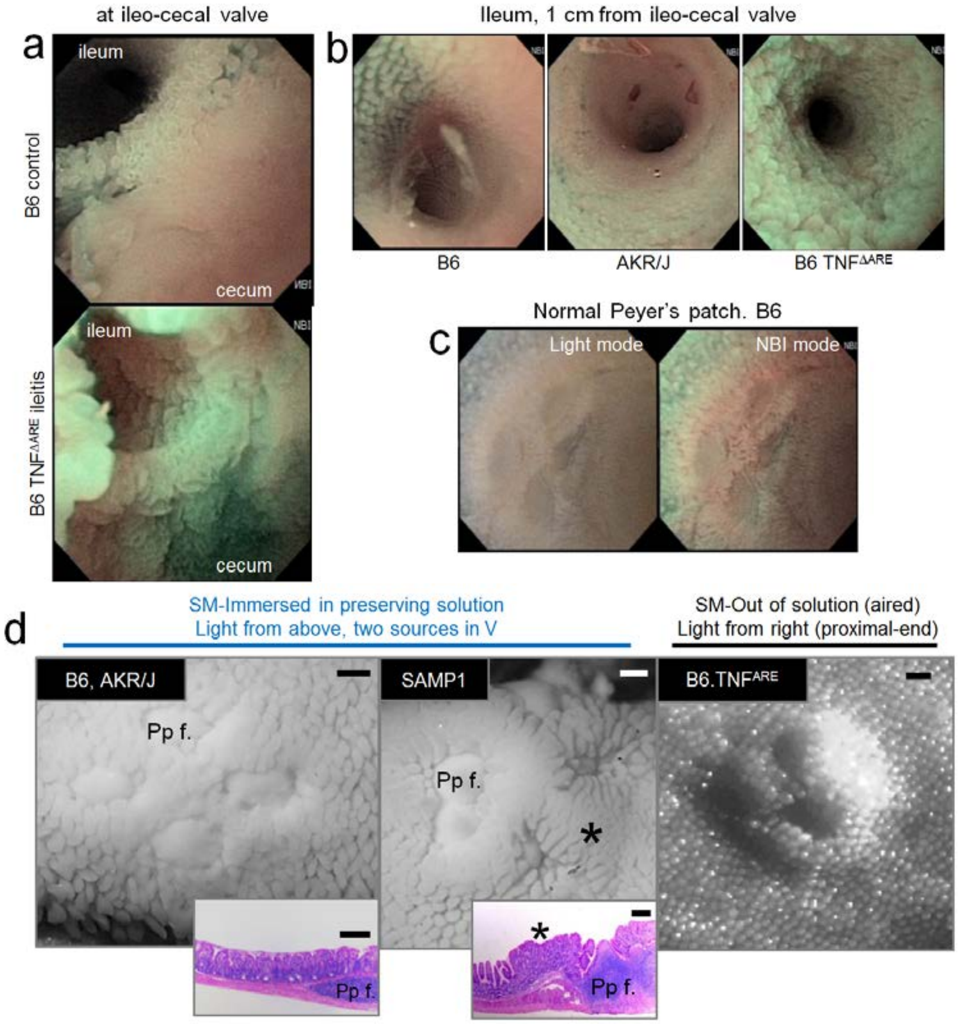

Supplementary Figure 2. Endoscopic examination of the ileum (ileoscopy) in B6$^{TNF\Delta ARE/+}$ (TNF^{ARE}) mice, and SM. (**a**) Ileo-cecal valve at 30 weeks of age. (**b**) Ileum 1 cm from cecum, note the enlarged villi. No cobblestones as shown in Fig. 1 were identified endoscopically. (**c**) Peyer's patch appearance in normal B6 mouse of panel 'a'. (**d**) Stereomicroscopic protruding appearance of Peyer's patches (Pp f., follicle) in SAMP mice compared to the typical thin appearance of Pp in healthy B6 and AKR, and in proximal segments of TNF^{ARE}. Images obtained with tissue immersed in 70% ethanol and with incident light from above, except TNF^{ARE}. Best SM contrast is achieved with aired (not soaked) tissues, and with proximal-to-distal ~300 angle incident light parallel to longitudinal intestinal axis (**Fig 1c** and **Supplementary Movie**). Notice TNF^{ARE} image has more 3D appearance. Histological sections of gut wall and Pp in ileitis-free and SAMP mice; Pp f, Peyer's patch follicle. H&E. Bar, 200 µm; Asterisk, cobblestones

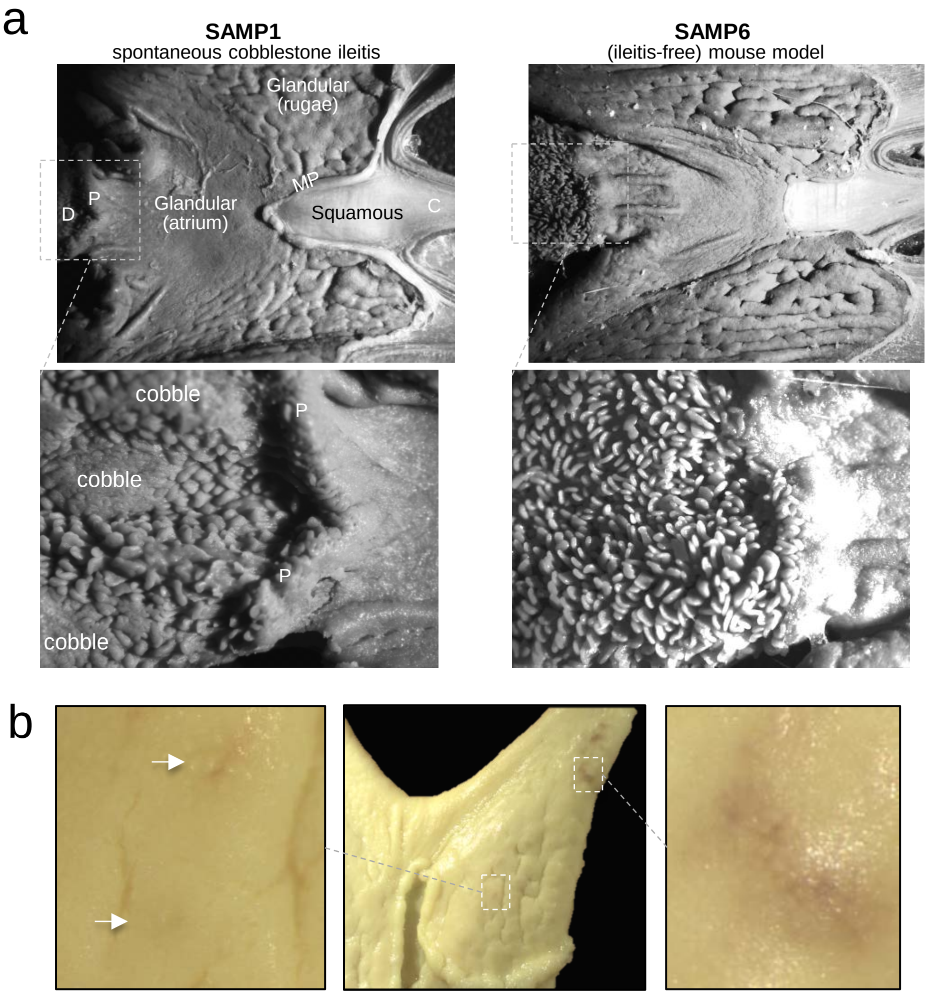

Supplementary Figure 3. SM examination of the stomach and proximal duodenum in mice. (**a**) SM en-face view of the gastric mucosa in mice allowed the discovery of cobblestone-like Crohn's disease lesions in the duodenum of SAMP mice, and differences in mucosal villous density and shape; and in the stomach, gastric glandular mucosa rugosity and thickening of the margo plicatus (MP). SAMP6 strain is used as an ileitis-free colitis-prone strain for comparison. SM shows the murine gastric mucosa is composed of squamous and glandular regions (atrium, smooth; and fundus, rugose), separated by the Margo plicatus (limiting edge). C, cardias near esophageal entry; D, duodenum, P, pylorus. (**b**) Color SM images to illustrate focal mucosal reddening (hemorrhages; arrows in left panel are faint due to fixation) in glandular mucosa of SAMP1 mouse, following Bouin's fixation. Blood vessel congestion/mucosal hemorrhages are best assessed in fresh tissues (**Supplementary Fig. 4**).

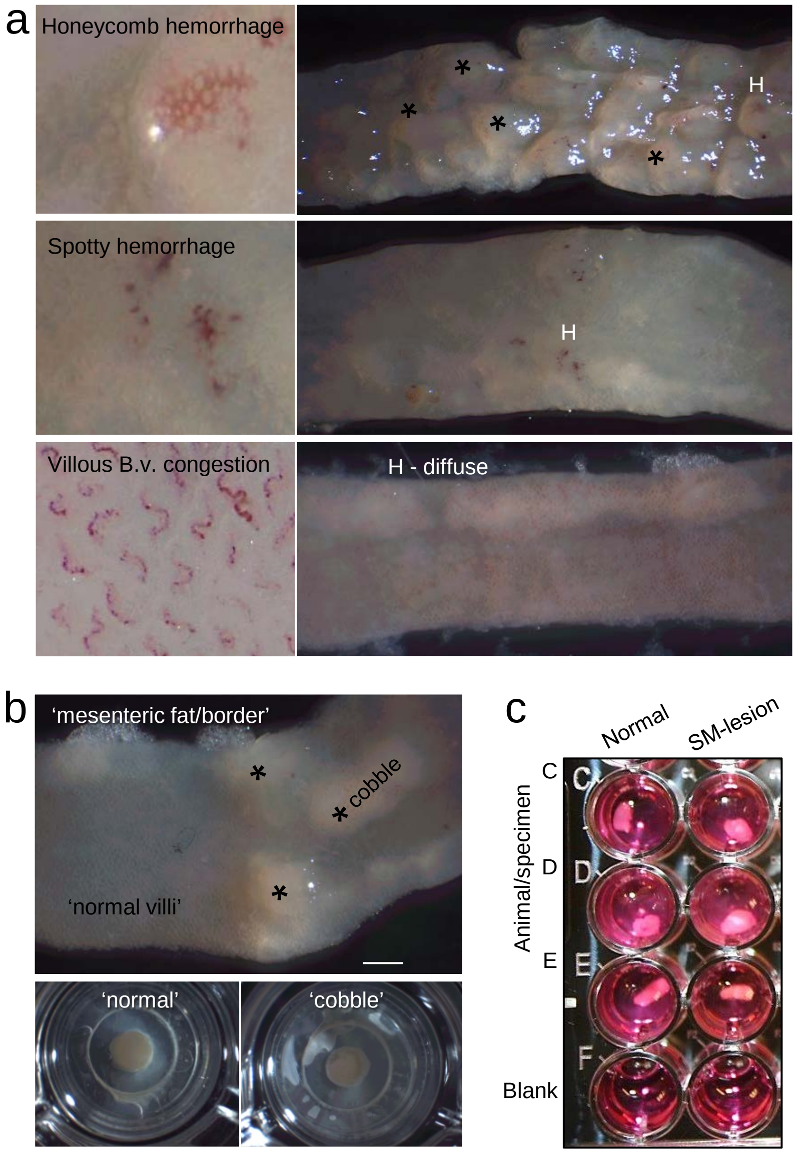

Supplementary Figure 4. SM analysis of fresh intestinal tissue allows the assessment of vascular congestion and the microdissection of areas of interest for functional analysis. (**a**) Vascular congestion changes in the intestinal tract of mice can be assessed and reported as described. Notice advanced cobblestones in top panel (asterisks). Scoring of SM lesions however is best accomplished after Bouin's fixation (see **manuscript**). (**b**) SM-dissection of fresh tissues for 'within-mouse paired-sample' functional analysis; this example illustrates the device wells set with SM-abnormal tissues for an ex-vivo transmembrane epithelial electric resistance (TEER) assay (see manuscript). (**c**) Another example of harvest of fresh tissue in specific 3D-SM-lesions for ex-vivo tissue culture assays for preclinical drug testing and protein and gene expression analysis.

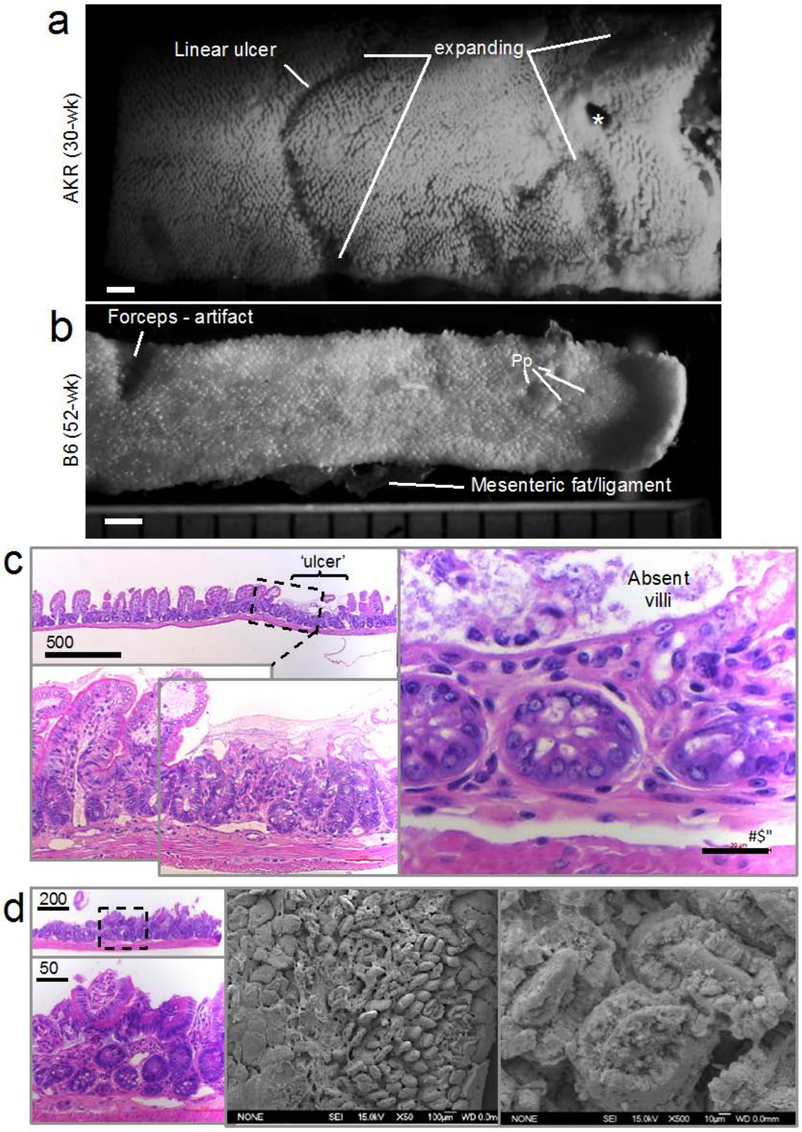

Supplementary Figure 5. Linear ulcers and villous erosion in the small intestine. Bouin's fixed ileum. (**a**) SM revealed the presence of linear ulcerations in ileitis-prone and ileitis-free mice; the cause and significance remains unclear. Note the correspondence of lesions on both sides of the specimen. Asterisk, pin hole print left by needle during tissue fixation. Bar, 1 mm. (**b**) Illustration of location of artifacts due to tissue forceps trauma to villi during tissue processing, with no correspondent on other edge of specimen. Pp, Peyer's patch. Bar, 1 mm. (**c**) Histology shows often these lesions are narrow in traditional longitudinal thin-sections. H&E, Bars in µm. (**d**) Scanning electron microscopy confirmed another type of SM abnormality: Villous erosion. More commonly found in animals with certain types of intestinal SM abnormalities, histology and SEM indicated that erosion of the tip of villi as seen with SM could be due to intercellular junction anomalies in the villous epithelium. The significance and cause is unclear. SEM images, notice the presence of various stages of villous distortion/erosion. Sample from a B6 mouse; harvested with SM for high-power SEM-target analysis/validation. SM allows the identification of concurrent focal pathologies, and various stages of 3D-SM changes or focal lesion expansion.

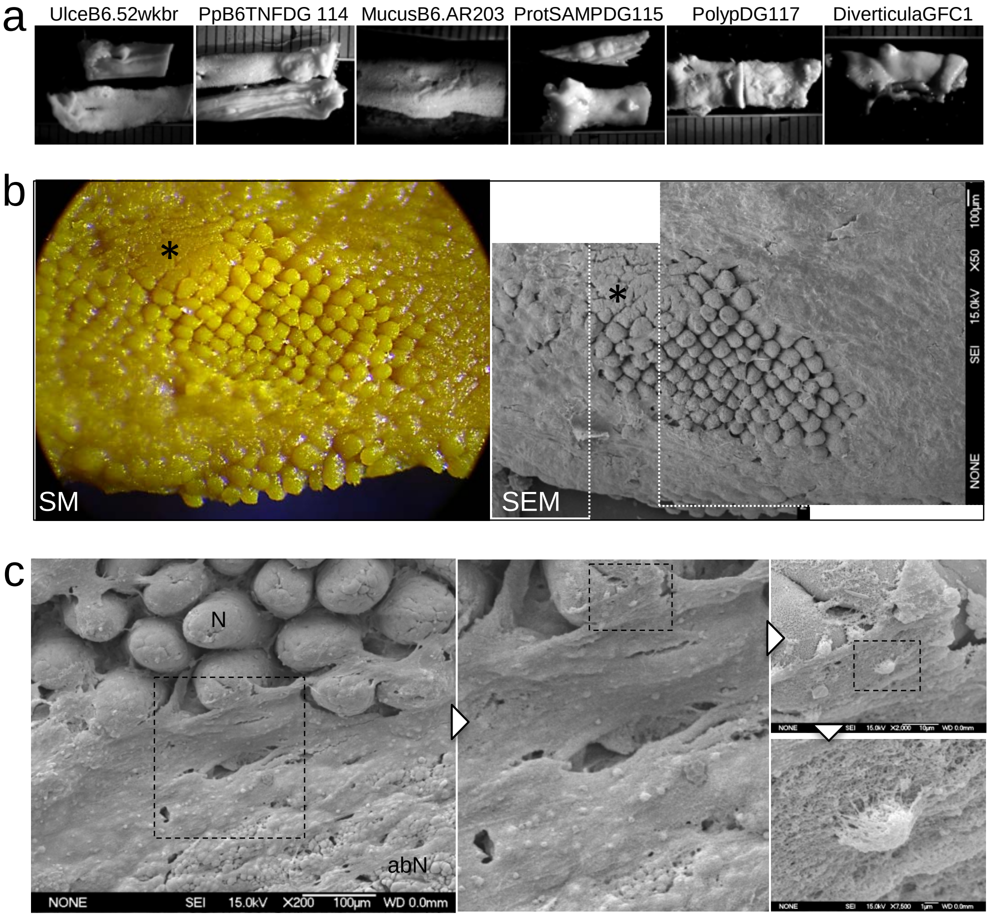

Supplementary Figure 6. Most mice affected with intestinal disease have at least another type of SM-lesion. (**a**) Examples of representative concurrent anomalies harvested for histology and SEM. Following histological and SEM validation, a catalog of lesions, for each large and small intestine, was created by grouping lesions to 10 scoring categories (see **manuscript**). (**b**) Side-by-side comparison of SM and SEM images of a specimen covered with another SM-abnormality: layers of muco-fibrinous material. Illustrating the advantages of (color) SM for rapid 3D-profiling of intestinal inflammation, notice that to achieve the example field-of-view with SM would require at least two perfectly aligned SEM images. SEM is technically-demanding, expensive, and is current use in intestinal research is not as popular as in past years (**Supplementary Fig. 1d**). Also note that semi-transparent tissues become opaque in SEM when covered with (gold) metal particles to allow electron reflection; thus, no structures can be inferred underneath those layers as shown. Asterisk, early cobblestone lesion. (**c**) SEM images of another specimen covered with muco-fibrinous material. Normally, healthy mice have intestinal villi that appear clean, but in seemingly affected mice, the mucosa can be irregularly covered with mucus/fibrin strands. Using high-power SM-target SEM analysis we determined that such layers often cover abnormal intestinal villi with altered morphology and increase gut luminal cellularity. Targeted SEM analysis would be highly valuable if SM was used to identify 3D-SM structure patterns of interest. N, normal villi; abN, abnormal villi.

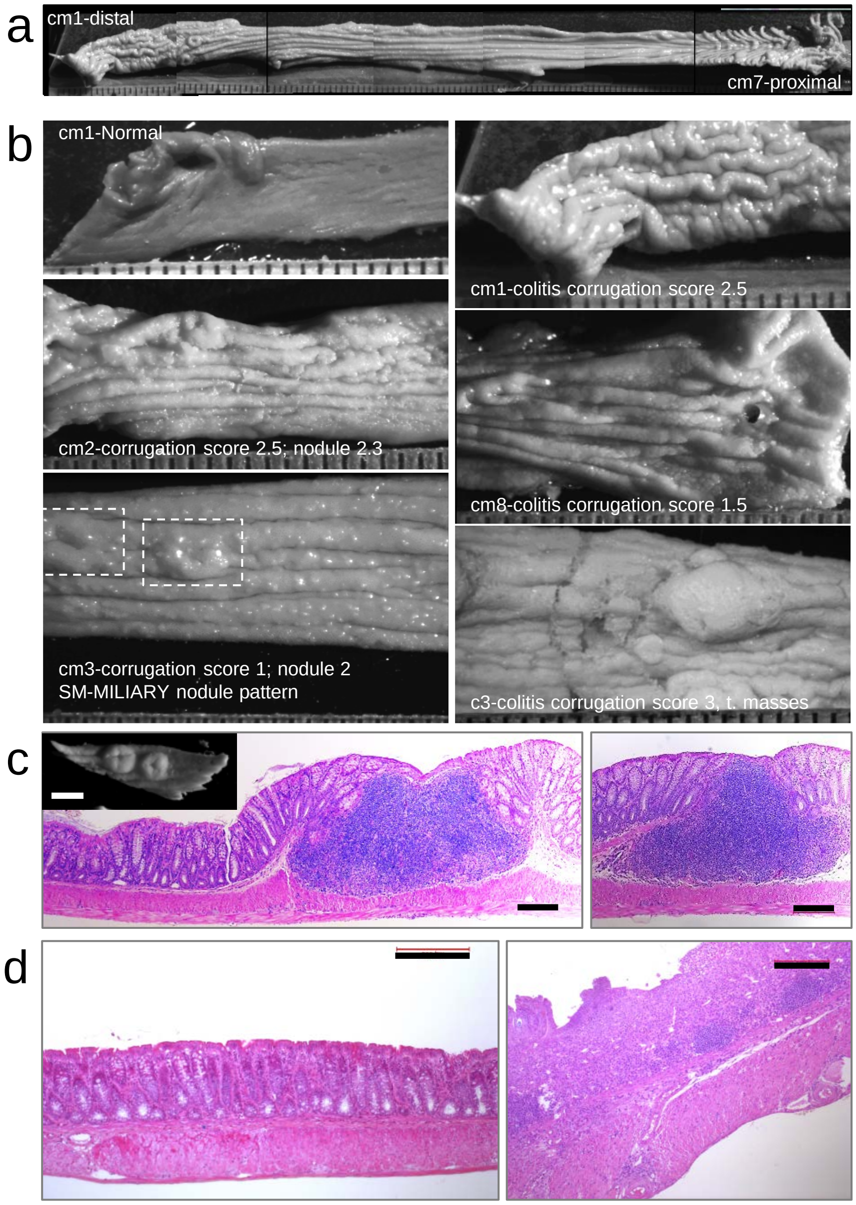

Supplementary Figure 7. SM patterns of colon corrugation, milliary pattern, lymphoid nodules and tumor appearance, and histology of the colon in mice. (**a**) Screenshots from SM-videos were used to assemble this colon image. Rapidly aligned in power point [mouse+Alt], then copied/pasted in Adobe Illustrator, image is suitable for ImageJ analysis (see sections below in manuscript). Notice corrugation and regional topographical variation. Ruler has 0.5-mm steps. (**b**) Examples of SM-3D-abnormalities not considered or quantified with traditional histology or histological scores. Dashed white frames illustrate common lesions in the colon of mice; histologically, they are aggregates of inflammatory mononuclear cells resembling lymphoid nodules. (**c**) Histological sections of ulcerated nodules of inset image (bar, 2 mm), representing the ones framed in panel b. Colonic epithelium is discontinued; resembling ulcerous nodules (aphthous-like ulcers)[2]. (**d**) Histological sections of normal and affected mid colon cut along their longitudinal axis. Notice that traditional (longitudinal) histological sections would be suboptimal to assess the 3D-SM patterns seen in panel b. Bar 200 µm.

3D-Stereomicroscopic Assessment and Pattern Profiling of Intestinal Inflammation in Mice*

(3D-SMAP*gut*)

SCHOOL OF MEDICINE
CASE WESTERN RESERVE UNIVERSITY

Date: __________ Stereo-request ID: [ ] Mouse ID: _______

Scored by: _______ Experimenter/experiment name:_____/____________

Treatment/days of treat.:__________/_____ Mouse strain & age: _____&___

colon – SM-corrugation score:

average _______ /cm

total

	1	2	3	4	5	6	7	8	9	10 cm
=	+	+	+	+	+	+	+	+	+	
Mucosal corrugation: Corr. severity= (II, x)										
% mucosal surface (100 / 50 / 0)										
Qualitative type of nodule/ulcer/ or mass, Up to 2/cm: type(n)										
type(n)										
Map of masses, proximal folds & special lesions										

Anus ——— Cecum

Maximum corr. | Average/cm

Type frequency: 1ry, 2ry, 3ry

Rank gut lesions based on count in all affected cm

Observations:

Decimal Score: ________ . ________ (corrugation s.) (decimals) .

Colon lesions – 3DSM severity:

(decimal identifiers)

Corrugation severity (choose higher to record):

0- smooth surface, subtle folds
1- Mild corrugation, like flattened waves
2- Moderate, "brain appearance"
3- Severe cobbles/ulcers;
4- Tumorous-like masses

Qualitative type of inflammatory structure/pattern

(Nodule/Tumorous mass with respect to ulceration):

t1-normal discrete/flat nodule covered with mucosa (lymphoid aggregates that can be seen in healthy mice)
t2-prominent nodule covered with mucosa
t3-large protruding nodule with large central ulcer
t4-large nodules with severe central and surrounding ulcerations (example severe DSS-colitis type)
t5-Protruding polyp or tumorous mass with distinctly abnormal mucosal crypt pattern (example AOM/DSS-tumorous masses)
t6-Other:___________

Special	
Photo	

ileum – SM % of abnormal mucosa and types of SM lesions:

	1	2	3	4	5	6	7	8	9	10 cm
Ileum lesions use abbreviation s Up to 3/cm: Lesion										
Lesion										
Lesion/villi										
% for each cm: Abnormal mucosa										
(normal mucosa)	( )	( )	( )	( )	( )	( )	( )	( )	( )	( )
Cobbles, total n=										
Peyer's Patch, type=										
Map protruding, masses, ulcers & special lesions.										

Cecum ——— jejunum

Lesion count, order (1, most common)

	Abb.	freq.	d.i.
1			
2			
3			
4			
5			

Average/cm Abn. Normal ( )

Total/average

Abn/N ratio:

Summary statistics:

Length of ileum examined: ____cm

OPTIONAL

Size of lesion (*% area due to lesion/n lesions*):

- 1ry Lesion Size: ______
- 2ry Lesion Size:______
- 3ry (or villi) Size:______

Decimal Score: __________ . ___ ___ ___ ___ ___ (% abnormal.m x10) (decimal identifiers) .

Observations:

MPO weigthed

Abnormal m. MPO (*U/g*): _____. Re-scaled MPO:_____ x % of abnormal m. ( )=_________**(abn)**

Normal m. MPO (*U/g*): _______. Re-scaled MPO:_____ x % of normal mucosa ( )=________ **(n)**

MPO-weighted-3DSMAP%score: (abn) + (n) = __________

Ileum lesions – 3DSM severity:

(decimal identifiers [d.i.] & abbreviations [Abb.])

Choose predominant & severe categories/cm, assign decimal unit.

0 – vN, Normal villi (finger like, tongue like, thin plates)**.**

1 – vBA, Villous blunting/angular villi; M_1, frequent mucofibrinous material strands between villi. Do not use to estimate %.

2 – $M_{2\text{-}3}$, mucofibrinous clusters that cover, or totally obscure villi visualization

3 – E, Erosion, villi tips appear eroded

4 – $U_{1\text{-}3}$, Ulceration, areas where clustered villi look devoided from epithelium cap or appear homogenously smaller than the surrounding villi. Linear ulcerations are common in certain mouse strains. Map ulcer shape in form.

5 – T, Villi aggregates as in TNF^{ARE} 3D-stereoenterotype. No cobblestones as in SAMP-3D-stereoenterotype.

6 – S, Stricture. Narrowing of mucosal perimeter. Detected before opening gut, or opened in assoc. with circumferential thickening of wall. Difficult to assess; best: observe during flushing with trans-illumination.

7 – $D_{1\text{-}3}$, Diverticulum. Cavity in intestinal wall covered with mucosa, and with/out feces. D1, <1 gut lumen diameter, D2, 1-2x lumen; D3, >2x wider than lumen, impacted, inflamed/discolored.

8 – $C_{1\text{-}3}$, Cobblestone villi aggregates C1, early; C2, moderate, C3, advanced

9 – $P_{1\text{-}3}$, Proliferating tissue like polyps or tumor-like masses. Not for artifacts.

NOTE: Severity can be assigned to each lesion as subscripts **1**-mild, **2**- moderate, and **3**-severe. (eg., C1, early or mild cobblestone)

Special	
Photo	

*Using digital identifiers: The scale developed for small intestine ranges from 0.000 to 1000.987. Score 0 means healthy. Score 456.789: an average of 45.6% of abnormal mucosa per linear cm of gut examined; decimals 789 indicate that the abnormality was due to lesion categories 7, 8 and 9 in order of decimal identifiers prevalence. For colon, which lack villi, the SM-colon corrugation score is simpler/faster to determine and considers the colon shortens during colitis in arbitrary scale. See article for details.

Supplementary Figure 8. Scoring form. Decimal numbers allow tracing and differentiation of structures and the use of parametric statistics. Here we used 3 decimal places for identification of lesions (see footnote in the pictured form); however, this format can be expanded to more decimal places to store information for downstream analytical purposes (see **Methods** and **Supplementary Figs. 9** and **10** for basic and optional concepts) and protocol settings for stereoscopic imaging, MPO and fecal flora homogenization[3].

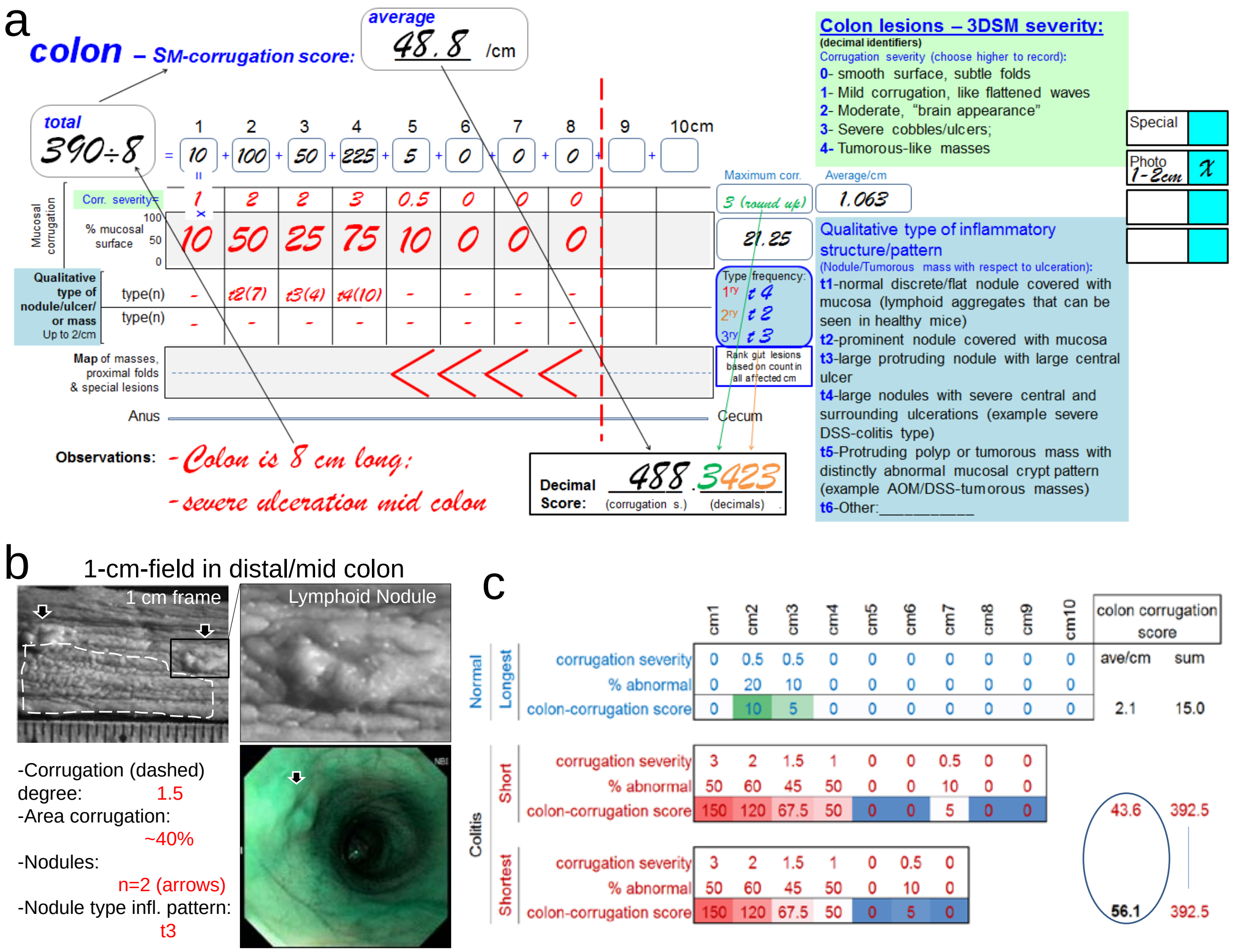

Supplementary Figure 9. Scoring example for the entire large intestine (rectum-to-proximal colon) of a mouse with DSS-colitis. (**a**) 3D-SMAP scoring form section with clinical data from a mouse after 7 days in 3%-DSS treatment. Notice *i)* the orientation of tissue is from left-to-right for distal-to-proximal (anus-cecum), *ii)* that the total score is the sum of the multiplication between the % of abnormal mucosal surface (corrugated or with other lesions) and the predominant corrugation severity, for each centimeter, and *iii)* that the colon is 8 cm long. Cursive fonts in red indicate three aspects assessed and recorded during SM assessment of tissues using the information boxes to the right (highlighted), and one aspect for mapping. The '<<<<' drawing symbolizes the mucosal folds of proximal colon, an aspect that cannot be easily discerned with 2D histology sections. The remaining numbers in black are calculations that can be optionally used for disease severity comparison. The cumulative 'decimal corrugation score' contains inherently information that can be easily decoded in downstream analysis, and further customized by adding more descriptors to the type lesion section (see 't6-Other'). (**b**) SM images illustrating how simple is to estimate the area of abnormal corrugation and the presence of ulcerated nodular (lymphoid) masses in the large intestine. Note the correspondence of SM nodule lesion appearance with that of endoscopic imaging (example in NBI mode, different mouse). Because the nodules appear aligned in a quarter of the transverse intestinal diameter, histology is more likely to miss those features with at most 1 in 4 chances of sampling the lesion, and even less probability to sample the masses at their center where they would be more informative. Further illustrations are available in accompanying protocol to aid in the assessment of the colon SM topography[3]. (**c**) Simulation examples for SM scoring rationale. As the colon shortens with DSS-colitis (primarily seen in mid/distal colon; while proximal colon is less affected), we determined that although animals could be compared using the total sum scores, the average/cm is more intuitive (max. value is 400; 100%*corrugation4), produces more Gaussian data distribution (total score sum data is skewed to the right), and reflects the inflammation driven-shortening (note 43.6 vs 56.1) for the colon making it more suitable for parametric statistics. The effect of inflammation on colon shortening has not been considered in histological protocols.

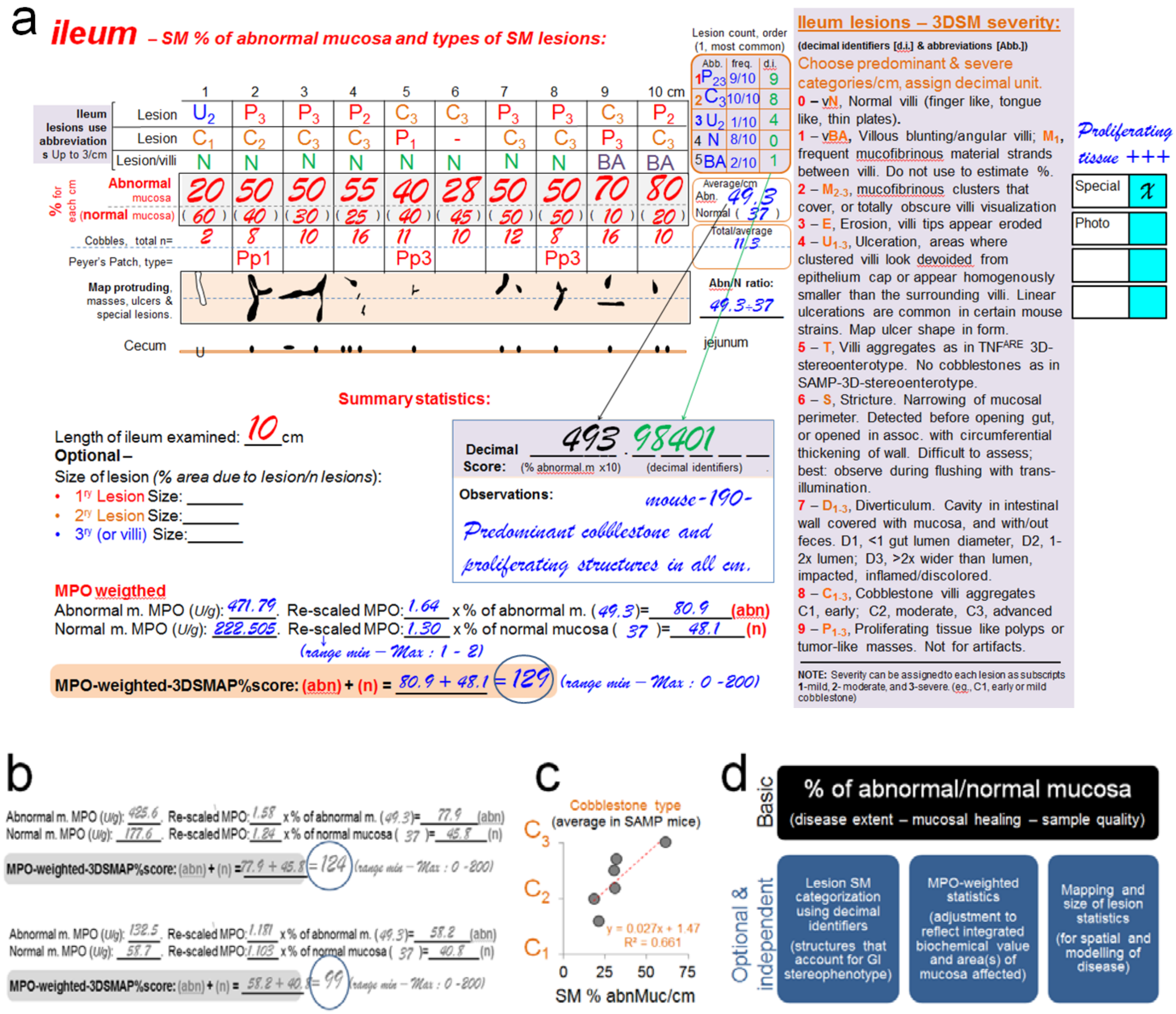

Supplementary Figure 10. Scoring example for the small intestine of a mouse with advanced cobblestone ileitis. (**a**) 3D-SMAP scoring form with data from a 30-week-old SAMP mouse maintained in SPF conditions with regular diet P3000. Note that the orientation of tissue is from left-to-right for distal-to-proximal (ileocecum-jejunum). The scoring system was designed to test the last 10 cm of small intestine, but it is also suitable for the entire intestinal tract in mice (20-30 cm)[3], or even for larger species. Ten centimeters were determined to be ideal for inter-mouse comparability in the case of diseases with regional variability (*e.g.*, experimental Crohn's disease); shorter fragments will introduce larger variability of data estimates within an experimental group. (**b**) Calculation examples to illustrate the magnitude of the MPO-weighted SM-%AbMuc data changes that could be observed for actual MPO data from two littermate mice assumed to have the same % of abnormal/normal mucosa. Although the presentation of (MPO and SM-%AbMuc) data in murine biology can be done separately as it is traditional in immunology, the analysis of data controlling for intra-mouse correlation is important (mandatory in epidemiology research). Because researchers may not be familiar with multivariable statistical methods, we combined the two parameters to weight the data within each mouse. Although, our MPO-SM is not intended to replace proper multivariable analysis required to control for 'interaction' and 'confounding' variables, our MPO weighted data is stringent, reducing the chance of reporting false-positive findings. (i.e., minimal differences that could yield a significant p-value in univariate analysis are less likely to yield significance with the weighted MPO-SM data). (**c**) Example of applicability of lesion severity (1-3): cobblestone type (severity) correlates with the extent of disease severity/ileitis (% of abnormal mucosa/cm). (**d**) Conceptual aspects that are part of the scoring system for the small intestine that can be complemented with options customizable/amenable to study the role of inflammatory 3D-structure on GI phenotype.

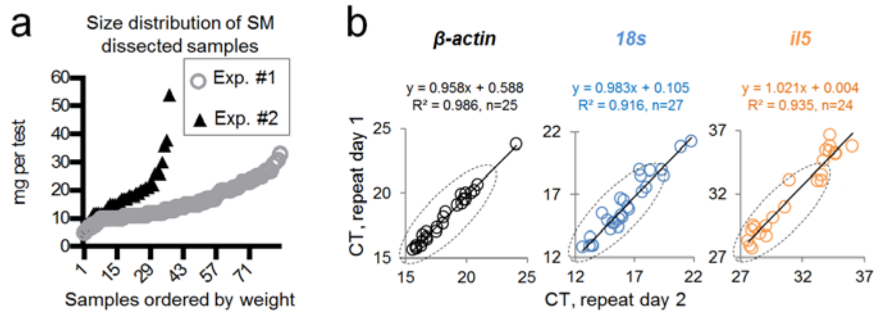

Supplementary Figure 11. Sample sizes (mg) of intestinal tissue SM-dissected from various animal models in this study. (**a**) Distribution of sizes from two separate experiments. Smaller samples often represent very 'precise dissection' strategies, while larger 'half- or complete-circumference' sampling strategies where tissues were locally homogeneous and had with minimal 3D-anomalies. (**b**) qPCR quantification of genes from random samples SM-dissected in this study tested in separate days to illustrate (SM, extraction, and qPCR) assay suitability. Note the data dispersion with respect to area shape shown for visual contrast (dashed oval).

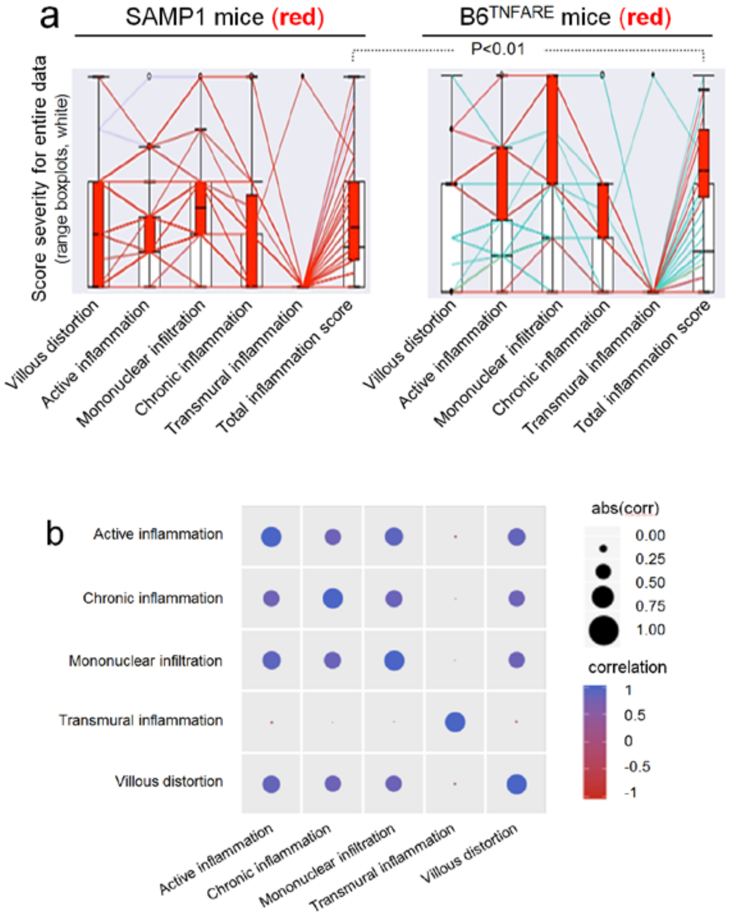

Supplementary Figure 12. Total histological inflammation scores used for ileum are based on five indexes; three of which we found to be highly collinear (redundant). Scoring was conducted in a blinded fashion for ileitis-prone and healthy mice (total=84 mice, 20-30 weeks-old). (**a**) Screenshots of interactive boxplot and line-analysis using public-domain Jaguar/R software (http://rforge.net/JGR/) of the histological inflammation indexes used in this study. The white boxplots represent the distribution of all data points (SAMP and TNF^{ARE}), while the red boxplot and lines highlight and contextualize the data distributions specific to either TNF^{ARE} or SAMP mice. Note that the total scores are significantly higher for TNF^{ARE}; and that the scores cluster at (integer) scale intervals, which is suboptimal for parametric statistics. With the exception of villous distortion, histology would misleadingly indicate that TNF^{ARE} is a better (more affected) IBD model for preclinical research purposes. (**b**) Correlation matrix analysis shows high correlation (r>0.9) and therefore collinearity among three of four indexes of inflammation (active, mononuclear, chronic; notice convex pattern in panel 'a') and the lack of correlation with transmural inflammation. These indexes are diagnostically redundant and do not convey information with respect to 3D-structure. Chronic inflammation scores would indicate that SAMP ileitis has less chronicity, but SM indicated that cobblestones are focal sites of presumptive perpetual chronic inflammation. Villous distortion scores are subjective and in TNF^{ARE} (adjusted p<0.001) they would indicate that TNF driven inflammation in B6 does not affect the mucosa. SM revealed however that each mouse strain has a unique 3D-SM structure pattern profile, both of which are completely different and therefore are highly relevant for preclinical and translational research in IBD.

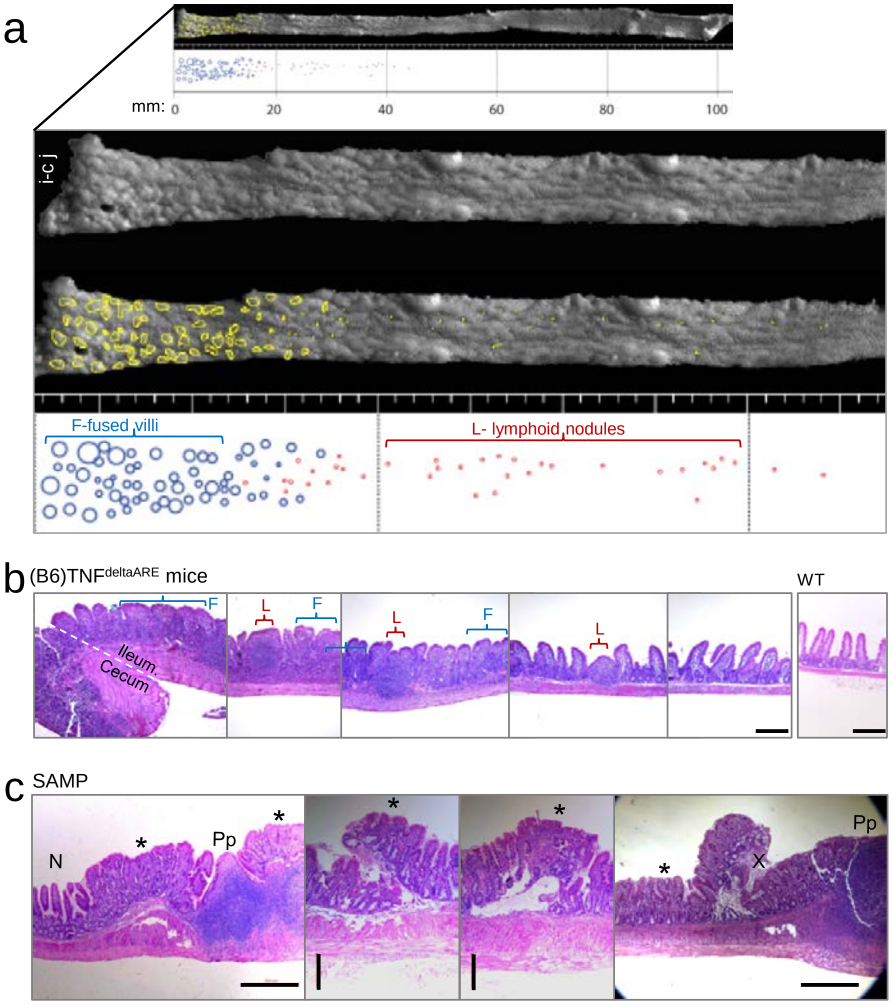

Supplementary Figure 13. Spatial and histological analysis of SM lesions in TNF^{ARE} mice. (**a**) This is the randomly-selected 30-wk old example depicted in **Fig 4a**. 3D-SMAP and histological analysis of seemingly different lesion types (F large blue circles; and L small red circles) indicated that TNF-driven ileitis has a proximad gradient pattern of chronic IBD progression that starts in proximity to the ileo-cecal junction (i-c j) in 100% of B6$^{TNFARE/-}$ mice. The mechanism of such pattern is unknown, but must differ from SAMP in which 50% of mice have no involvement of tissue in proximity to the cecum (**Fig. 4b**). (**b**) Thin-sections of representative mice as a function of distance from the cecum (left). Note the correspondence of villi fusion and shortening described in **Fig. 1c** and the image above. (**c**) Thin-sections of representative cobblestone lesions (asterisks) in SAMP mice for comparison. SM revealed that with marked expanding villous fusion, 'tissue protrusion' of the cobblestone edges can be seen (1-5 / mice at 30 weeks of age), in some cases with ensuing histological morphological alterations (x; notice this picture was taken using transillumination and the stereomicroscope). Tissue protrusions are rare events in TNF^{ARE} mice. Examination of fresh and post-fixed tissues indicates (polyp-like) tissue protrusions are not tissue-fixation artifacts. Pp, Peyer's patches; N, normal villi. H&E, Bar 200 µm.

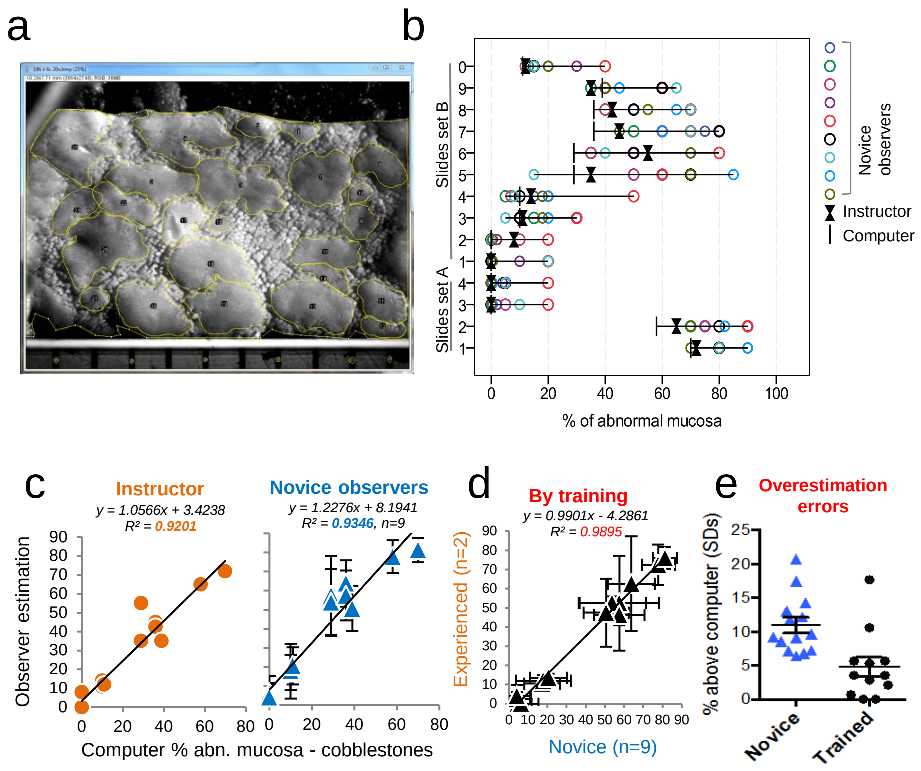

f

Individual performance and variability of 9 examiners after scoring 14 SM images

Examiner	R^2	Slope	Intercept	level of experience
	Coeff of variation	trend with computer	% overstimation vs computer	
**Perfect:**	1.000	1	0.0	**referent - computer**
P1	**0.949**	1.1748	**0.5**	novice
P2	**0.931**	1.1864	2.5	novice
P3	**0.920**	**1.0566**	3.4	**instructor**
P4	0.896	1.3919	4.2	novice
P5	0.882	1.3149	11.2	novice
P6	0.859	1.3676	4.4	novice
P7	0.829	1.3934	7.3	novice
P8	0.829	**1.0254**	26.7	novice
P9	0.805	1.2013	5.6	novice
P10	0.778	1.1328	7.5	novice

Supplementary Figure 14. A training session to estimate the 3D-SM% of abnormal mucosa affected with cobblestone lesions indicates that 3D-SMAP*gut* could be rapidly implemented by nonspecialists. (**a**) Example of a power point slide prepared for two training session sets. Following exact computer estimation of % of abnormal mucosa due to cobblestones in the ileum using Image J, individuals were trained by asking them to estimate areas affected by cobblestones, with/without the perimeter yellow lines. Cobblestones used for training since they were easy to identify among other SM-lesion categories. (**b**) Actual percentage estimates for slides presented, compared to computer and instructor estimates. (**c**) Correlation plots between computer measures and human estimates for instructor and trainees. (**d**) Correlation plots between human estimates for two types of trainees. Novice with 10 minutes of training vs individuals with at least 5 hours of supervised experience. (**e**) Most of the high variability observed among novice trainees, was due to overestimation of % of abnormal mucosa, which improves with training. (**f**) Correlation predictive models estimated for each trainee with respect to computer measurements. With basic training some individuals made highly accurate estimations. With experience, the assessment of 10 cm of intestine may to take as little as 60 seconds with healthy tissues, or as long as 10-15 minutes each in cases of complex 3D-IBD patterns.

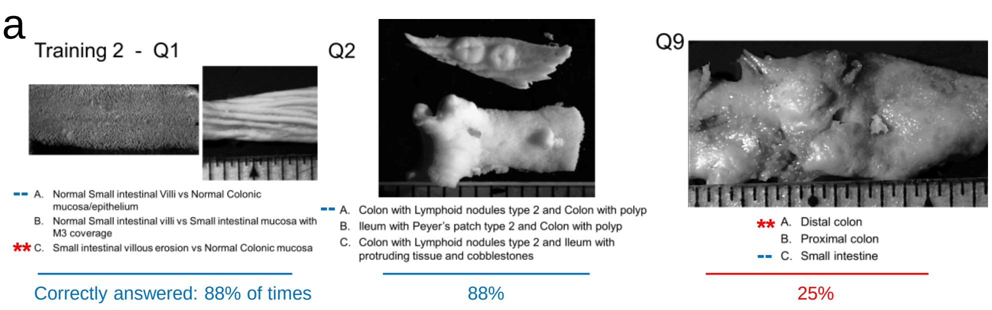

b

Observer: GI research experience	Previoius hours of SM training	S10	S12	S1	S2	S3	S4	S6	S8	S13	S5	S11	S7	S9	performance sum	performance %
5y	Training 1 + 3	1	1	1	1	1	1	1	1	1	1	0	1	1	12	92.3
Tech	Training 1 + 0	1	1	1	1	1	1	1	1	1	1	1	0	1	12	92.3
2y	Training 1 + 0	1	1	1	1	1	1	1	1	1	1	1	1	0	12	92.3
2y	Training 1 + 0	1	1	0	1	1	1	1	1	1	1	1	1	0	11	84.6
2y	Training 1 + 0	1	1	1	1	1	1	1	1	0	1	1	0	0	10	76.9
Tech	Training 1 + 0	1	1	1	1	1	1	0	1	1	0	0	1	0	9	69.2
None	0	1	1	1	1	0	0	1	1	1	1	1	0	0	9	69.2
None	0	1	1	1	0	1	1	1	0	1	1	1	0	0	9	69.2
	sum	8	8	7	7	7	7	7	7	7	7	6	4	2		
	Correctly answered %	100	100	87.5	87.5	87.5	87.5	87.5	87.5	87.5	87.5	75	50	25	Mean	80.8
		less difficult										more difficult			SD	10.9

Slides with multiple-choice question (correct answer, 1; wrong, 0)

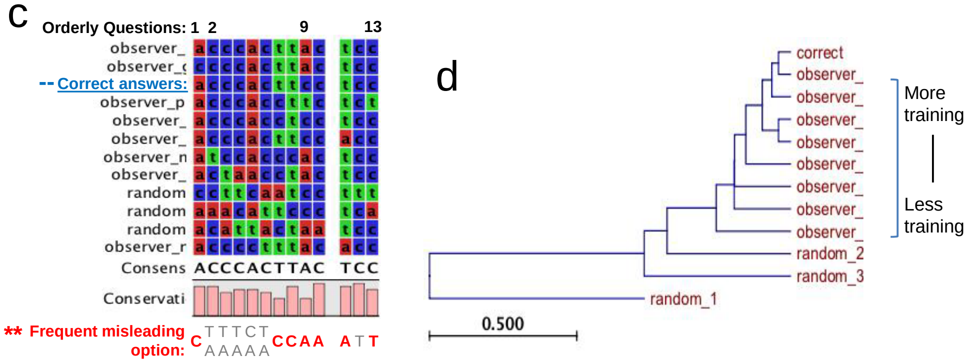

Supplementary Figure 15. A second training session quantified the observer's ability to differentiate among the catalog of SM lesions in large/small intestinal SM images. (**a**) Example of power point slides prepared for testing 8 observers after a 40 minute training session in which the principles for SM differentiation and intestinal anatomy were highlighted. Thirteen questions (Q) were immediately formulated using multiple choice answers to allow observers to engage in a non-timed comparative analysis process using the catalog of SM lesions described in the **Methods** section and **Supplementary Fig. 8** as reference material. Notice the complex number of variables that must be taken into consideration to answer a given question. --, correct; **, frequent misleading option (see below). (**b**) Ranking of observers and questions based on test performance and training experience. The circled area shows difficult questions were answered by observer with more hours of training. (**c**) For cluster analysis purposes, the answers provided (A, B, C letters) were replaced by A, T, and C letters to use hierarchical sequence cluster analysis principles to determine the frequent cause of assessment mistakes (we use DNA sequence algorithm and free software CLC Sequence Viewer 6). This analysis highlights what type of mistakes were common, allowing training reinforcement on certain SM lesion types. Notice the context of misleading options in panel 'a'. Multiple choice questions can be expanded to four letters (ABCD > ATCG) to enable the use of nucleotide analysis as observers gain experience or to add one more distractor. (**d**) MPGA analysis illustrates observers' sequential performance (Q1-Q13) in the context of random responses.

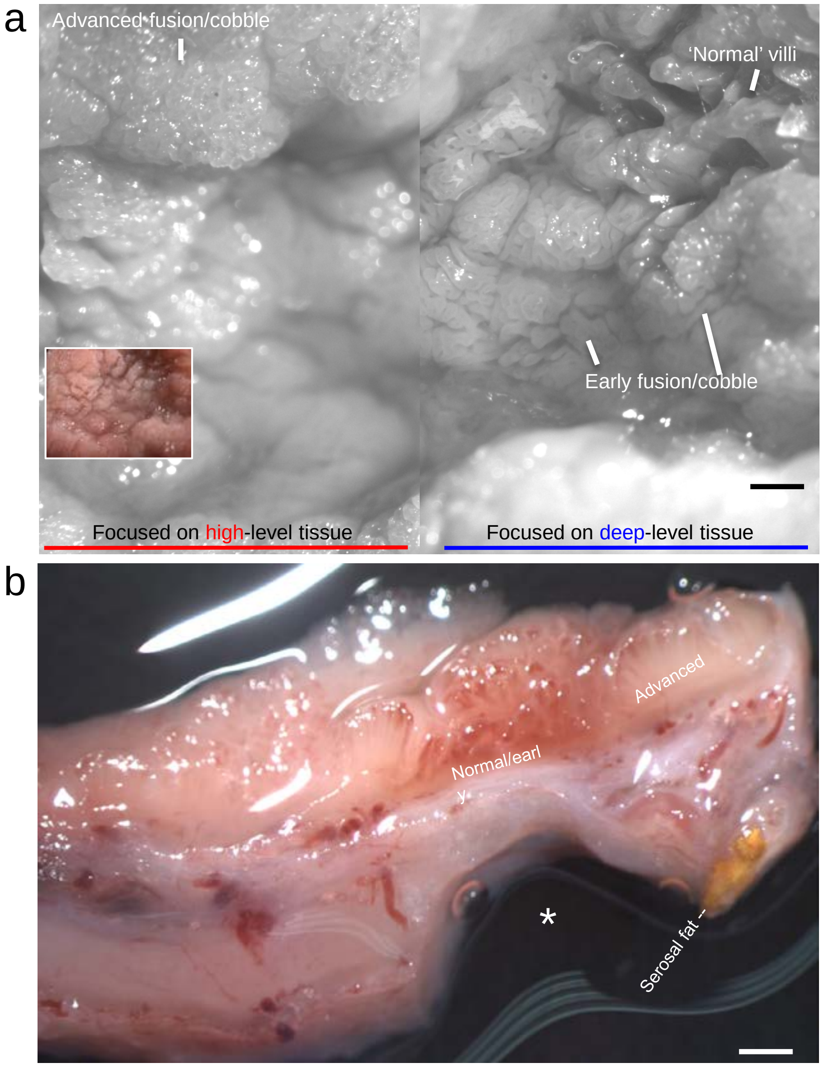

Supplementary Figure 16. ***En face* and transmural SM examination of 70% ethanol-moistened ileal mucosal specimen affected with Crohn's disease.** Formalin-fixed. (**a**) Magnification of 'inset *en face* image' depicted in **Fig. 6f.** Notice the three distinct stages of villous aggregation/fusion in sample: normal, isolated finger-like villi; early, villi fuse and form convoluted sheets of villi; advanced, villous appearance is lost resembling more a homogeneous flattened surface. (**b**) Subsequent transmural examination of the same specimen moistened with 70% ethanol. Notice differences on villi congestion in areas that correspond to advanced villous fusion (cobblestones), and normal-villi/early-villous-aggregation. Notice sub-mucosal vessels and serosal mesenteric yellow fat. *, artificial space created during resection of tissue by pathologist prior to SM examination. Bar, 1 mm.

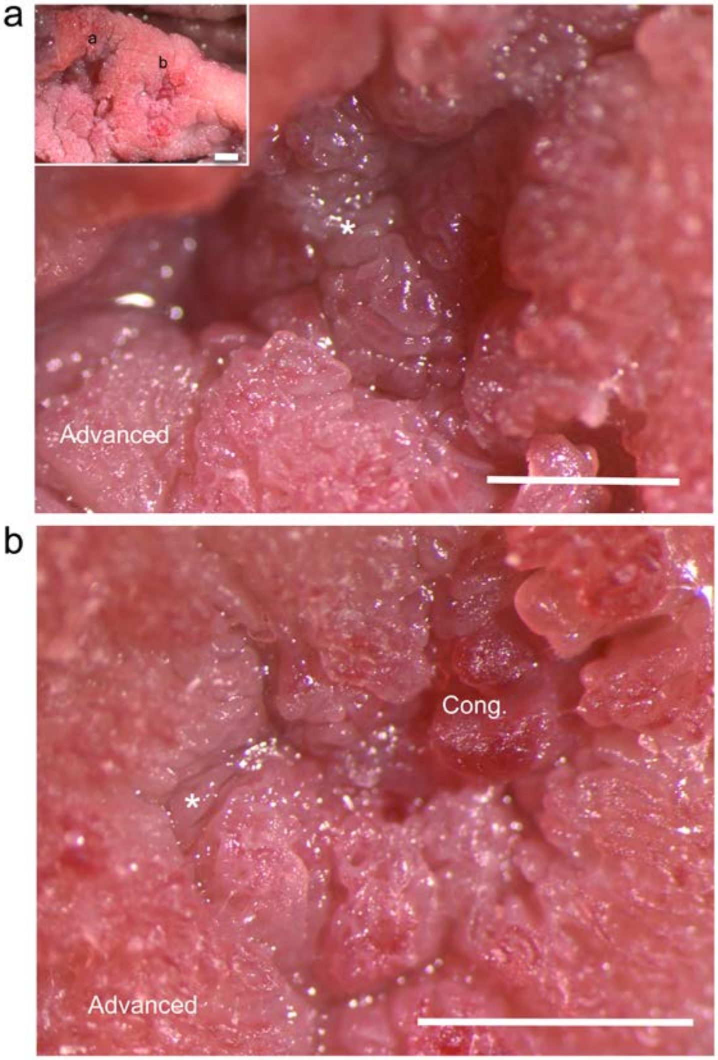

Supplementary Figure 17. ***En face*** **SM examination of severely affected area of ileum with Crohn's disease following evaporation/diffusion of moistening ethanol.** Formalin-fixed; villous details can be best observed during the transition from moistened to dry. (**a**) Inset highlights location of two depressed small areas on severely affected ileal mucosa surrounded by advanced areas of villous fusion ('Advanced' cobblestones). Cong., congested villi. (**b**) Small depression 'b' from inset in top panel shows the villi at the center appears congested and surrounded by severely fused villi. en face examination of mucosa indicates ileal cobblestones in CD expand concentrically. *, early villous involvement/fusion. Bar, 1 mm.

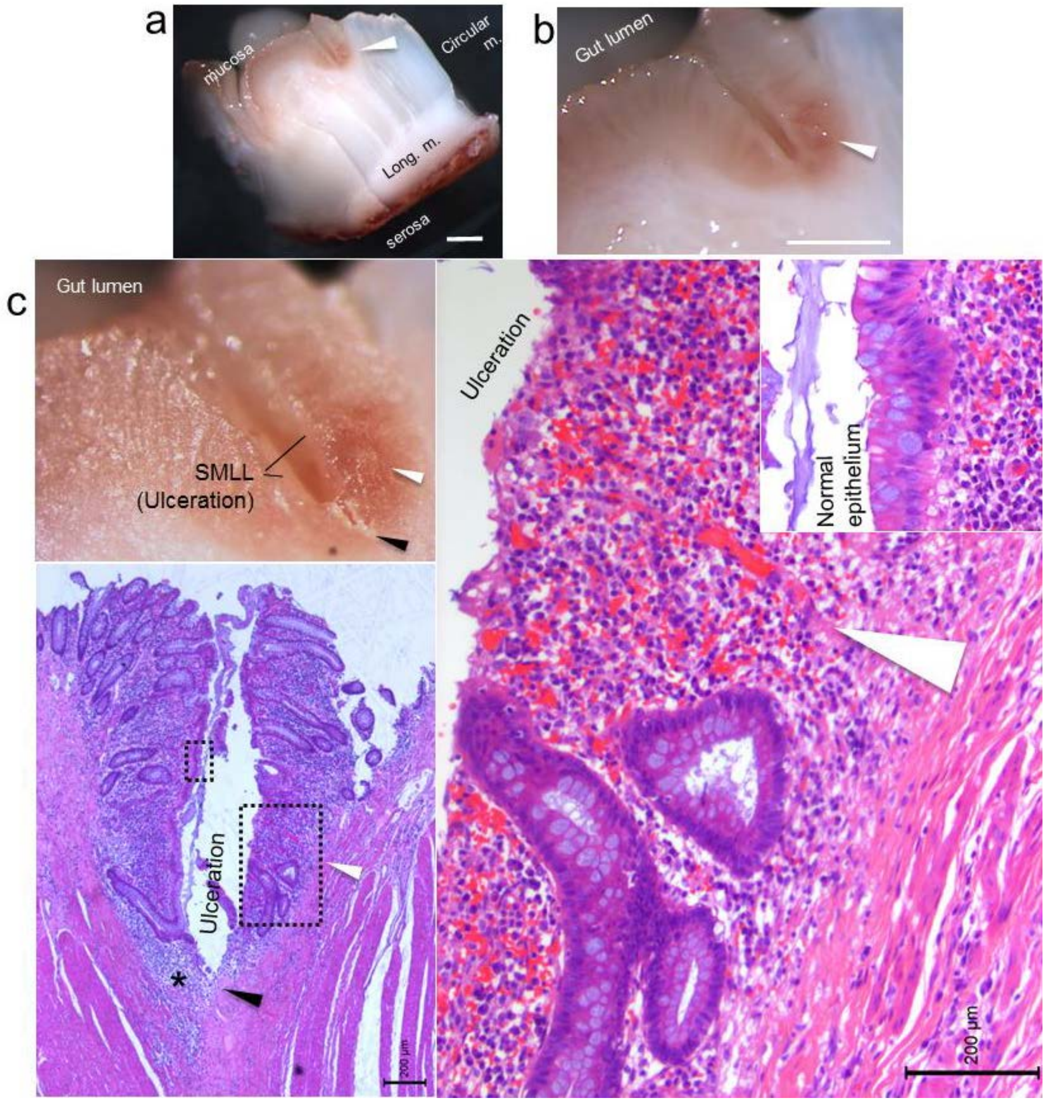

Supplementary Figure 18. Transmural SM examination of area of colon deemed normal/nonaffected in surgical specimen from patient with Crohn's disease. Formalin-fixed. (**a**) Block of tissue (4mm x4mm x 4mm) harvested at random. Notice gut layers and area of congestion in mucosa (arrow). Bar, 1mm. (**b**) Close-up at congestion site (white arrowhead) with tissue moistened with 70% ethanol. (**c**) Magnification at SM-congestion lesion with tissue aired, that is, not soaked with ethanol. White arrowhead, vascular congestion, SMLL, SM-liquefaction lesion indicative of ulceration; black arrowhead, fissure with abnormal semi-purulent debris; asterisk, marked inflammatory cell infiltrate in site of fissure (see proposed terminology description in **Supplementary Table 1**). Note the correspondent histological inflammatory congestive reaction at the site identified sites and the presence of ulceration. Inset, normal mucosa in site opposite to ulcerated mucosa shown. With histology, SM has the potential to improve the selection and harvest of inflamed and noninflamed tissues for less-variable focal epigenetic/gut flora studies. The collection of mucosal samples based on SM-appearance is now feasible and ideal.

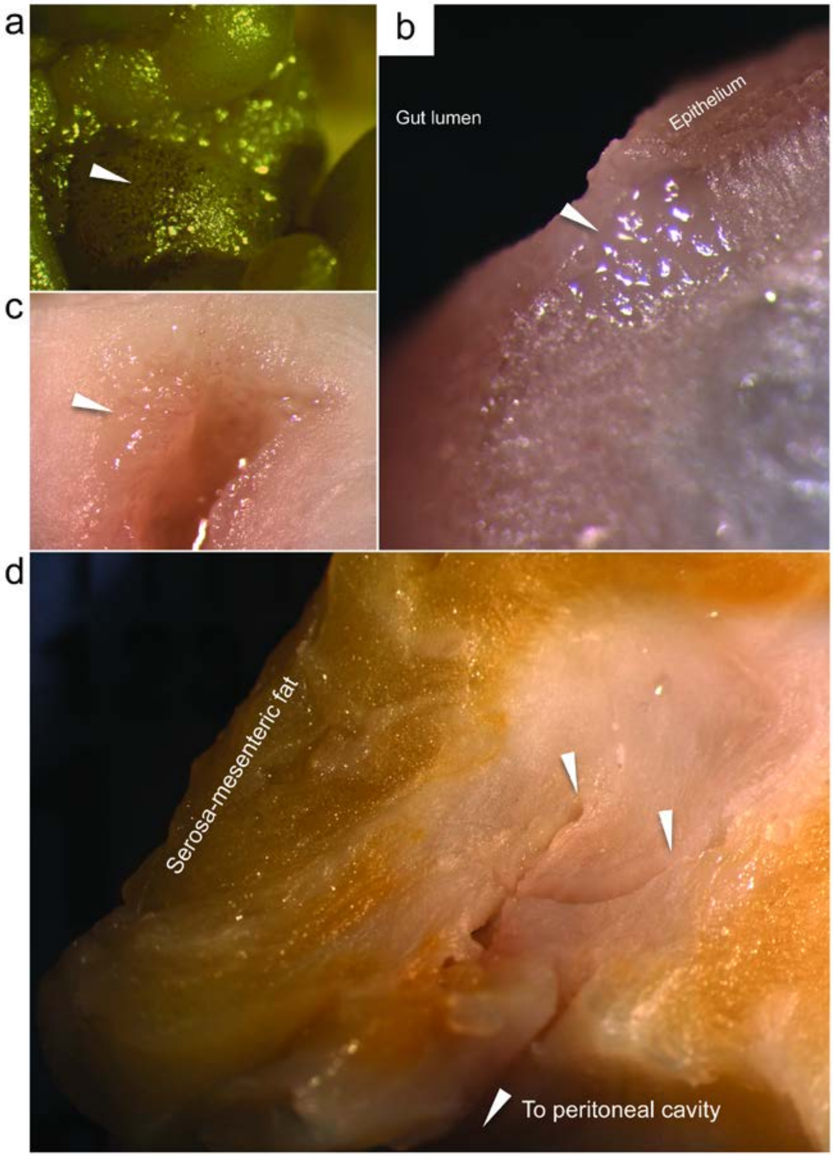

Supplementary Figure 19. ***En face*** **and transmural SM examination of severely affected colon with Crohn's disease revealed lesions that could contribute to increase intestinal permeability in 'leaky gut' conditions.** (**a**) *En face* mucosal surface; notice cobblestones, lack of villi, and hemorrhages in epithelium (arrow). (**b**) Colonic mucosa with focal area of SM-liquefaction (arrow; glossy appearance). (**c**) Magnification of cavernous fistulous tract (CavFT) in muscular layer of colon depicted in **Fig. 6a**-**inset** after removing the pus by gentle flushing. Notice liquefaction appearance of the CavFT lining surface. (**d**) Another sample section from specimen in **Fig. 6a** magnified, emphasizing the presence of abnormal serosa and the presence of CavFT connecting the fistulous complexes from the muscle layers (arrows) to the serosa/peritoneal surfaces. SM enabled the precise and rapid microdissection of structure lesions for 16S microbial analysis (**Fig. 6e**). Notice that 'cobblestones' in colon differ in nature of formation from that of the ones in the ileum.

Supplementary Table 1. Stereomicroscopic transmural profiling of intestinal specimens: terminology.

**SM-cobblestone (SmCobbles).** Aggregated villi in the small intestine or bulking lesions in the colon, corresponding to the equivalent cobblestone lesions observed during endoscopy in humans, can be also referred to as cobblestones in stereomicroscopy.
**Mucosal fissures (SmFissures).** Lesions that correspond to cracks in the epithelium accompanied with areas of abnormal discoloration or texture in the mucosa/submucosa areas that correspond to the confluence of two cobblestone lesion curvatures.
**SM congestion lesions (SmCL).** SmCL are areas with high red discoloration and visible blood vessels compared to surrounding tissue, similar to SmLL (see below) discoloration when tissues are moistened with 70% ethanol, but that appear dry upon evaporation of the ethanol. SmCL may represent areas of early inflammation with predominant vascular congestion (and no tissue liquefaction as SmLL).
**SM liquefaction lesions (SmLL).** Resemble areas of 'melting' (liquefaction) of tissues whose solid structure disappears due to inflammation or tissue degradation. Areas have red discoloration and contain more visible blood vessels. Upon dehydration of formalin-fixed ethanol-preserved tissue, SmLL lesions maintain a moistened appearance with respect to their surroundings, suggesting physico-chemical tissue property changes resulting from inflammation. SmLL represent areas where severe infiltration of immune cells has replaced matrix structure and matrix forming cells. They contain higher cellularity (host nuclear DNA copies) if determined by B-actin qPCR CT values on DNA extracts (not RNA/cDNA). SmLL affect any gut tissue layer.
**Penetrating fistulous tracts (PFT).** Linear irregular-edge lesions that resemble narrow tracts running perpendicular to the gut lumen alongside muscle bundles of the circular muscle layer. They run from (sub)mucosal layers to reach the deep longitudinal muscle layer. The PFT may represent the earliest form of dissecting inflammatory conditions that communicate (sub)mucosal layers with deeper structures such as muscular or serosa layers.
***Cavernous* Fistulous tracts (CavFT).** Lesions with irregular cavitating appearance resembling caves. In cavitating lesions the normal tissue is replaced with a cavity. CavFT may contain purulent material and are >1mm width. FCavTs that travel deep between the circular and longitudinal gut muscle layers can be designated intramural (IM)-FCavT. Stereomicroscopic analysis of serial sections indicated IMCavFT lesions travel parallel to the gut lumen and have long-range spreading potential along the gut longitudinal axis; no capsule structures typical of abscesses were observed.

Supplementary Table 2. Host and 16S bacterial primers used in this study.

GENE	Forward (5'-3')	Reverse (5'-3')
rn18s	ACGGAAGGGCACCACCAGGA	CACCACCACCCACGGAATCG
sdha	GGAACACTCCAAAAACAGACCT	CCACCACTGGGTATTGAGTAGAA
mpo	AGGATAGGACTGGATTGCCTG	GTGGTGATGCCAGTGTTGTCA
tlr4	ATGGCATGGCTTACACCACC	GAGGCCAATTTTGTCTCCACA
β-actin	CAGGGTGTGATGGTGGGAATG	GTAGAAGGTGTGGTGCCAGATC
il1b	TCCTTAGTCCTCGGCCAAGAC	GTGCCATGGTTTCTTGTGACC
il5	GCTTCTGCACTTGAGTGTTCTG	CCTCATCGTCTCATTGCTTGT
Universal	TCCTACGGGAGGCAGCAGT	GACTACCAGGGTATCTAATCCTGTT
Bifidobact	GCGTGCTTAACACATGCAAGTC	CACCCGTTTCCAGGAGCTATT
Lactobacilli	AGCAGTAGGGAATCTTCCA	CACCGCTACACATGGAG
Prevotella	CACCAAGGCGACGATCA	GGATAACGCCYGGACCT
Sfilb779/1008	TGTGGGTTGTGAATAACAAT	GCGGGCTTCCCTCATTACAAGG
sfilb1380	N/A	GGTTAGCCCACAGGCTTCGG
bacterioides	285-GGTTCTGAGAGGAAGGTCCC	Eub355-GCTGCCTCCCGTAGGAGT
Clos-I	TACCHRAGGAGGAAGCCAC	GTTCTTCCTAATCTCTACGCAT
CloXIV	GAWGAAGTATYTCGGTATGT	CTACGCWCCCTTTACAC

References supporting the bacterial primers have been previously validated for qPCR quantification[4-10]. Validated mouse primers were obtained from http://pga.mgh.harvard.edu/primerbank/

Supplementary References

1. P. K. Nighot and A. T. Blikslager, ClC-2 regulates mucosal barrier function associated with structural changes to the villus and epithelial tight junction *Am J Physiol Gastrointest Liver Physiol* **299** (2), G449 (2010).
2. A. F. Ravens, Inflammatory bowel disease--be aware of the mimics. Diagnosis: lymphogranuloma venerum *Gut* **55** (3), 333 (2006).
3. A Rodriguez-Palacios and F Cominelli, Stereomicroscopy and target myeloperoxidase intestinal phenotyping *Protocol Exchange* (2015).
4. Y. Yin, Y. Wang, L. Zhu, W. Liu, N. Liao et al., Comparative analysis of the distribution of segmented filamentous bacteria in humans, mice and chickens *ISME J* **7** (3), 615 (2013).
5. A. Couturier-Maillard, T. Secher, A. Rehman, S. Normand, A. De Arcangelis et al., NOD2-mediated dysbiosis predisposes mice to transmissible colitis and colorectal cancer *J Clin Invest* **123** (2), 700 (2013).
6. N. Larsen, F. K. Vogensen, F. W. van den Berg, D. S. Nielsen, A. S. Andreasen et al., Gut microbiota in human adults with type 2 diabetes differs from non-diabetic adults *PLoS One* **5** (2), e9085 (2010).
7. X. W. Huijsdens, R. K. Linskens, M. Mak, S. G. Meuwissen, C. M. Vandenbroucke-Grauls et al., Quantification of bacteria adherent to gastrointestinal mucosa by real-time PCR *J. Clin. Microbiol.* **40** (12), 4423 (2002).
8. M. Haarman and J. Knol, Quantitative real-time PCR analysis of fecal Lactobacillus species in infants receiving a prebiotic infant formula *Appl. Environ. Microbiol.* **72** (4), 2359 (2006).
9. Y. Song, C. Liu, and S. M. Finegold, Real-time PCR quantitation of clostridia in feces of autistic children *Appl. Environ. Microbiol.* **70** (11), 6459 (2004).
10. M. T. Suzuki, L. T. Taylor, and E. F. DeLong, Quantitative analysis of small-subunit rRNA genes in mixed microbial populations via 5'-nuclease assays *Appl. Environ. Microbiol.* **66** (11), 4605 (2000).
